# How membership in the North Atlantic Treaty Organization transforms public support for war

**DOI:** 10.1093/pnasnexus/pgad206

**Published:** 2023-07-05

**Authors:** Michael Tomz, Jessica L P Weeks, Kirk Bansak

**Affiliations:** Department of Political Science and Stanford Institute for Economic Policy Research, Stanford University, Stanford, CA 94305, USA; Department of Political Science, University of Wisconsin–Madison, Madison, WI 53706, USA; Department of Political Science, University of California, Berkeley, CA 94720, USA

**Keywords:** international relations, military alliances, public opinion, survey experiments

## Abstract

How do military alliances affect public support for defending targets of aggression? We studied this question by fielding an experiment on 14,000 voters in 13 member countries of the North Atlantic Treaty Organization (NATO). Our experiment involved a hypothetical scenario in which Russia attacked a target country. We randomly varied the identity of the target (Bosnia, Finland, Georgia, or Sweden), and whether the target was a member of NATO at the time of the attack. We found that voters in every member country were far more willing to use military force to defend each target when the target was in NATO, than when the target was outside the alliance. The expansion of NATO could, therefore, transform European security by altering the likelihood and scale of future wars. We also uncovered important heterogeneity across targets: the benefits of joining NATO were considerably larger for Bosnia and Georgia than for Finland and Sweden, since most voters in NATO countries would defend Finland and Sweden even if they remained outside the alliance. Finally, the effect of NATO was much stronger among voters who perceived NATO as valuable for their own country. Rhetorical attacks on NATO could, therefore, undermine the alliance by eroding the public's willingness to defend other members, whereas rhetoric highlighting the benefits of NATO could bolster defense and deterrence. These findings advance knowledge about the effects of alliances, while also informing policy debates about the value and size of NATO.

**Significance Statement**

Some scholars argue that alliances can induce countries to fight wars they would otherwise prefer to avoid. Others contend that countries can easily disregard alliances when war would be inconvenient. To advance this important debate, we conducted a large-scale experiment. We found that voters in 13 member countries of the North Atlantic Treaty Organization (NATO) were far more willing to use military force to defend a country after it joined NATO, than to defend the same country before it joined NATO. Joining would help some countries more than others, however, and rhetorical attacks on NATO could undermine the alliance. Our findings have profound implications for understanding the effects of NATO, the Russian invasion of Ukraine, and the consequences of expanding NATO.

## Introduction

Observers have long debated the effects of military alliances on decisions to use military force to defend other countries. Some argue that alliances can induce countries to fight when they would otherwise prefer not to get involved. As such, alliances could widen the scale and intensity of wars, but they could also deter potential aggressors from instigating conflict in the first place. Others contend that without a global authority to enforce international agreements, alliances are mere scraps of paper that countries can disregard when war would be inconvenient. Indeed, studies have documented a surprisingly large number of cases in which countries chose not to honor their alliance commitments (1–4).

This debate is not only central to theories of international relations; it is also of practical importance for military planning, decisions about alliance membership, and the prospects for international peace. The Russian invasion of Ukraine in 2022 inspired Finland and Sweden to seek membership in the North Atlantic Treaty Organization (NATO), and Bosnia and Georgia have long been in the membership queue, but it is unclear how accession by these countries would affect the security landscape.

Resolving these academic and policy debates requires a deeper understanding of how alliances affect decisions about war. Given that countries have latitude to renege on their alliance commitments, it is important to consider how domestic political factors such as public opinion influence decisions to fight or not. Although leaders are ultimately responsible for foreign policy decisions in democratic countries, political scientists have long demonstrated that policymakers are responsive to and constrained by public opinion, particularly when it comes to highly consequential policy areas (5, 6). More specifically, growing evidence shows that, in democracies, public opinion influences decisions about military conflict (7–10), including decisions to defend allies (11–13). Moreover, meta-analyses have found that citizens and elites respond to political situations in “strikingly similar ways,” implying that surveys of ordinary citizens can reveal how decision-makers would think about political issues, even absent public pressure (14). Little research, however, has tested how alliances move public opinion, and previous work has focused solely on the United States (15, 16).

In this study, we advance knowledge on three dimensions. First, we investigate how alliances shape public opinion not only in the United States but in 12 other NATO members. Studying responses across NATO's membership is critical for informing theory and policy about the world's most powerful alliance. Second, we investigate whether the security benefits of joining NATO would be larger for some applicants than for others. Specifically, we compare the potential gains for Bosnia, Finland, Georgia, and Sweden, the four countries other than Ukraine that were furthest along in their bids for NATO accession at the time of our study. Finally, we test whether willingness to help NATO members depends upon how valuable voters a priori believe NATO to be for their own country. If so, political rhetoric disparaging NATO could undermine its ability to deter foreign aggression, whereas efforts to improve public perceptions of NATO could strengthen the alliance's effectiveness.

Estimating the effects of alliances is difficult with historical data because states do not form alliances randomly. We therefore fielded a large-scale preregistered survey experiment (17–19) on more than 14,000 voters in 13 NATO countries (Czech Republic, Denmark, France, Germany, Greece, Hungary, Italy, the Netherlands, Norway, Poland, Spain, the United Kingdom, and the United States), with our sample data reweighted to match key demographic margins from each country's population.

**Fig. 1.**
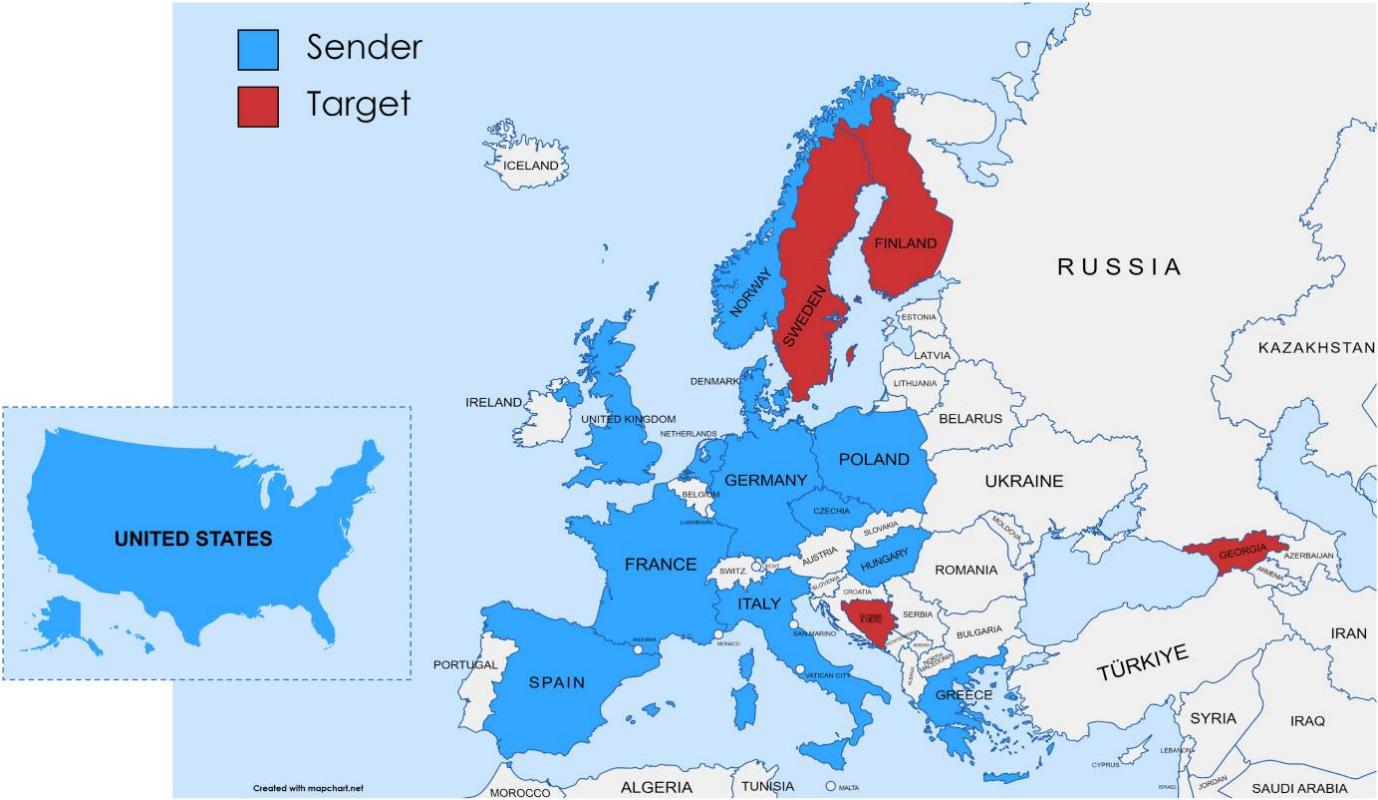
Map of sender and target countries in study. Figure displays the four possible target countries in the survey experiment scenario and the 13 sender countries (i.e. the survey respondents’ countries).

Our experiment described a hypothetical Russian attack on four possible *targets*: Bosnia, Finland, Georgia, or Sweden. We randomly varied whether the target was characterized as a NATO member at the time Russia attacked and measured whether voters thought their own country (the *sender*) should defend the target militarily. Figure 1 displays the sender and target countries in our study. We fielded our study during Russia's invasion of Ukraine and just before the 2022 NATO summit during which Sweden and Finland were formally invited to join the alliance—a moment when both interstate war in Europe and NATO expansion were top of mind, endowing the survey scenario with realism. Full details about the sample, design, and analysis can be found in the Materials and methods section.

We preregistered three hypotheses. First, all else equal, voters would be more willing to use military force to defend target countries if those countries were members of NATO, versus if they were not. In theory, alliances such as NATO could create reputational incentives to intervene. Failing to aid an ally could hurt one's reputation as a desirable security partner, jeopardizing current and future alliances (20–25). Alliances could also give rise to moral obligations. Mounting evidence shows that moral considerations influence public thinking about foreign policy (18, 26–31). Having promised to defend members of the alliance, citizens may feel an ethical duty to act. Finally, alliances could provide simple cues or cognitive heuristics to uninformed voters about whether to intervene (32–34).

The alternative perspective is that alliance treaties have little effect on public support for the use of force. Given the high costs of military intervention and no international authority to enforce international promises, voters in the United States and Europe might assign little weight to NATO membership when deciding whether to defend new alliance members against Russian aggression.

Second, we hypothesized that joining NATO would matter more for Bosnia and Georgia than for Finland and Sweden. As we detail in the Online Supplementary Material, Bosnia and Georgia are viewed as less democratic than Finland and Sweden; the costs of defending Bosnia and Georgia would likely be higher than the costs of defending Finland and Sweden, due to differences in military power, economic wealth, and compatibility with NATO's force structure; and for current NATO members, the economic and security consequences of a Russian attack would be lower if the target were Bosnia or Georgia than if the target were Finland or Sweden. Based on previous research in the United States (15), these are all reasons to suspect that willingness to defend Bosnia and Georgia in the absence of a NATO commitment would be lower than for Finland and Sweden, and hence that the effect of joining NATO would be larger for Bosnia and Georgia than for Finland and Sweden.

Third, we hypothesized that the effect of the target joining NATO should be largest among voters who think their own country's membership in NATO is a good thing. One might think that, by engaging reputational and moral concerns that transcend short-term material interests, alliances would have the same effects whether or not voters believe the alliance contributes to their own country's immediate security. We argue, however, that voters who value their own country's alliance membership should be even more sensitive to the negative consequences of failing to help an ally. Thus, alliances should more sharply increase support for war among voters who a priori prize the alliance than among those who doubt its value.

## Results

**Fig. 2.**
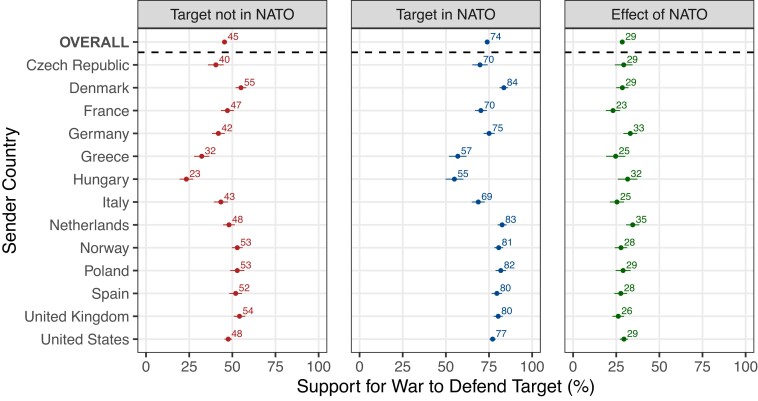
Effect of target joining NATO, overall and by sender country. Figure shows the percentage of voters who supported defending the target after a Russian attack, depending on whether the target was in NATO (middle panel) or not (left panel). The right panel shows the effect of the target being in NATO. Each row corresponds to a specific sender (i.e. respondent) country, with the top row showing the average across all sender countries. 95% CI are displayed.

To test these hypotheses, we designed our experiment to identify the effect of joining NATO, independent of other factors, among voters in a large proportion of NATO countries. Consistent with our first hypothesis, NATO membership powerfully shaped public support for war. Figure 2 depicts the percentage of voters who supported defending the target after a Russian attack, depending on whether the target was in NATO or not. The Materials and methods section provides details about our preregistered estimation procedures. The first row, which averages over both senders and targets, shows that when the target country was not in NATO, only a minority of voters across the 13 sender countries (45%) supported military intervention. Support swelled to an average of 74%, however, when the target joined NATO. This 29-point swing in public opinion is substantively sizable and easily distinguishable from zero.

The remaining rows of Fig. 2 display effects for each sender, averaging across targets. Baseline levels of support for defending non-NATO targets varied across countries. Nevertheless, in every sender country, the effect of NATO was large—between 23 and 35 percentage points. In summary, voters did not treat alliances as mere scraps of paper that could be dismissed when inconvenient, nor did they opt to “pass the buck” to other alliance members. Instead, alliances powerfully shaped public support for war.

**Fig. 3.**
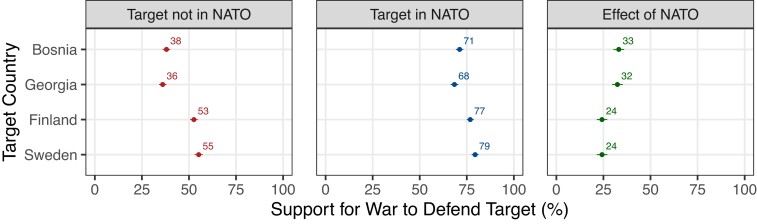
Effect of target joining NATO, by target country. Figure shows the percentage of voters who supported defending the target after a Russian attack, depending on whether the target was in NATO or not, with the results broken down by target countries (and averaged over sender countries). 95% CI are displayed.

Our experiment also supported our second hypothesis that joining NATO would matter more for Bosnia and Georgia than for Finland and Sweden. Figure 3 shows that only a minority of voters (36–38%) would defend Bosnia/Georgia when those countries remained outside NATO. Support nearly doubled, surging by 32–33 points, when Bosnia/Georgia joined NATO. Thus, the alliance commitment transformed skepticism into clear majority support. Support also increased when Finland and Sweden were characterized as NATO members, but NATO mattered less for these countries, which most voters (53–55%) would defend even without a NATO commitment.

The Online Supplementary Material further disaggregates these findings by sender. When we described Bosnia or Georgia as not in NATO, majority support for intervention emerged in only one country. Russia could, therefore, attack Bosnia or Georgia without fearing widespread public pressure for military involvement by NATO nations. In contrast, when we portrayed Bosnia or Georgia as part of NATO, majorities in 12–13 of our NATO sender countries favored intervention. Again, NATO membership was less consequential for Finland and Sweden, which majorities in 9 of 13 sender countries would defend even without NATO membership.

**Fig. 4.**
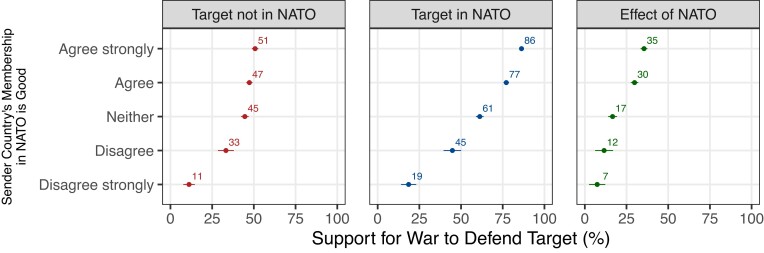
Effect of target joining NATO, by attitudes about NATO membership. Figure shows the percentage of voters who supported defending the target after a Russian attack, depending on whether the target was in NATO or not, with the results broken down by the respondents’ views on NATO (i.e. whether their own country's membership in NATO is good). The results are averaged over sender and target countries. 95% CI are displayed.

Finally, the experiment confirmed our third hypothesis that the effect of the target joining NATO should be larger among voters who value their own country's membership in NATO. Figure 4 separates voters into five groups, reflecting how much they agreed or disagreed that their country's NATO membership was a good thing (measured before administering the experiment). Among voters who agreed strongly, the effect of the target joining NATO was 35 percentage points. As enthusiasm for NATO waned, the effect of NATO declined in tandem. However, NATO had a significant effect even among voters who strongly doubted the value of their own country's membership, perhaps because they felt reputational or moral reasons to comply despite disliking the treaty. The Online Supplementary Material confirms that these conclusions also held after controlling for a wide range of respondent characteristics that are upstream of pro-NATO attitudes and might have contributed to the treatment effect heterogeneity in Fig. 4.

We conducted a series of exploratory analyses to test whether the effects of NATO depended on other attributes of respondents. Remarkably, the Online Supplementary Material shows that alliances had large effects regardless of left–right ideology, gender, or (in the United States) political party (Figs. S8–S15). Further, alliances mattered at least as much among individuals with “elite-like” characteristics such as high education, income, political interest, or being at least 40 years old (Figs. S16–S19).

The Online Supplementary Material also contains additional complementary analyses. Our findings held when we measured support for war on a five-point scale, chose not to weight the data, or both (Figs. S1–S7). Moreover, the Online Supplementary Material shows additional results of fielding our experiment in three *non*-NATO countries—Austria, Sweden, and Switzerland—alongside our main study (Figs. S20 and S21).

## Discussion

Our findings have important implications for understanding the effects of NATO and the consequences of NATO expansion. First, our experiments revealed that NATO can have powerful and widespread effects on public opinion, even though voters weigh many factors when evaluating war. We found that the target joining NATO produced a considerable surge in support for intervention for every combination of sender and target. Majorities almost always supported defending fellow NATO members, even when support for defending the exact same target would be in doubt without a NATO commitment. Although our experiment was not designed to identify the precise mechanisms through which NATO membership influenced public support for war, it seems plausible that many citizens were motivated to support targets out of concern for reputation, ethical duty, or both.

The expansion of NATO could, therefore, transform European security. On the one hand, expansion of NATO could increase the scale and intensity of future wars. Aggression against a country that had joined NATO would galvanize public support for war among allies in Europe and North America, potentially drawing a larger number of countries into war than an attack on the same country if it had remained outside the alliance.

On the other hand, expansion of NATO could make wars in Europe less likely. Our study highlights an important reason why NATO deters aggression. By increasing public support for retaliation, NATO membership makes the threat of retaliation more credible. Potential aggressors may be dissuaded from attacking, due to the expectation that an attack would trigger retaliation by other alliance members.

Second, our findings highlight the distinction between the *reliability* of alliances (1–3, 35) and their consequences. Reliability refers to the probability that a country will uphold its alliance commitment by defending an ally that has been attacked. Consequences refer to probability that a country will defend an ally, minus the probability of defending an otherwise identical nonally: in other words, how much does the alliance increase the likelihood of intervention? One might think that reliable alliances are consequential, and vice versa, but our findings underscore that reliability and consequences are distinct (36). Indeed, there may be an inverse relationship between reliability and effectiveness, with alliances less consequential when they are more reliable and less reliable when they are more consequential.

Consider, for example, how reliability and consequences varied across targets in our study. When Russia attacked a target that had joined NATO, public support for honoring the alliance was higher when the target was Finland or Sweden than when the target was Bosnia or Georgia. This pattern suggests that a NATO commitment to Finland and Sweden would be more reliable than a commitment to Bosnia and Georgia. At the same time, the effect of NATO membership on public support for intervention was larger for Bosnia and Georgia than for Finland and Sweden. Thus, in our experiment, NATO was more consequential when it was less reliable, and vice versa.

Our findings further imply that the impact of NATO enlargement should vary, depending on which countries join. We found relatively high public support for defending Finland and Sweden, even when those countries were not NATO members. Potential aggressors might, therefore, be deterred from attacking Finland or Sweden, regardless of whether those countries are in the alliance or not. In contrast, an aggressor could currently invade Bosnia or Georgia without fearing widespread support for retaliation, but support for intervention would skyrocket if these countries joined NATO. Thus, expanding NATO to include Finland and Sweden might be less consequential than inviting Bosnia and Georgia to become members. These findings have major policy implications for European security, especially since Bosnia and Georgia have both declared their aspirations to join NATO and have both been targets of external aggression in recent decades.

Finally, our findings suggest that the feelings voters have about particular alliances shape the defensive and deterrent value of those alliances. The alliance effect in our experiment was much larger among NATO supporters than among NATO skeptics. Rhetoric disparaging NATO could, therefore, weaken its influence (32, 37): if voters doubt NATO's value, they will be less motivated to take costly actions that sustain the alliance. And if potential aggressors believe that electorates are unwilling to defend allies, the threat of retaliation will become less credible, emboldening aggressors to attack (38–40).

This conclusion puts past criticisms of NATO—as well as broader critiques of institutions—in a new light. A range of political actors have engaged in anti-NATO political rhetoric, including extreme parties in Europe (such as the National Rally in France and Die Linke in Germany), former US President Donald Trump, and Russian leaders and operatives (41). Moreover, recent studies have shown that elite rhetoric affects public opinion about the benefits and obligations of alliances (32, 42). Our findings suggest that if criticisms of NATO are persuasive, they could erode the public's willingness to defend NATO allies and thereby encourage adversaries to attack with less fear of consequences. Conversely, our findings suggest that rhetoric highlighting the benefits of NATO could bolster defense and deterrence, even if an alliance does not expand. Thus, our study also provides an additional mechanism through which rhetoric can reassure alliance partners (43).

Our findings open many questions for future research. We found that NATO membership transformed public support for war. There are, of course, other major alliances involving other countries and other parts of the world. We expect that those alliances, like NATO, would increase public support for defending victims of aggression. Future research could explore whether other alliances, including ones with different contractual provisions (12, 23, 44), would affect public opinion.

One could also design experiments to test whether decisions to use force by some members of an alliance would cause other members to join the effort or instead lead them to freeride on the military efforts of other states. In addition, scholars could design studies to parse the mechanisms (e.g. reputation, morality, and cues) through which alliances shape public opinion and test whether those mechanisms are more powerful in some circumstances than in others. Future research could build on our experimental template to explore these important questions.

## Materials and methods

### Sample

We fielded a large-scale survey experiment in 13 NATO member countries: the Czech Republic, Denmark, France, Germany, Greece, Hungary, Italy, the Netherlands, Norway, Poland, Spain, the United Kingdom, and the United States. We also conducted a parallel study in three non-NATO countries: Austria, Sweden, and Switzerland. The interviews took place in May–June 2022.

For each European country, the survey firm Respondi sampled ∼1,000 adult respondents from the population of eligible voters. Respondi used country-specific gender and age quotas to recruit samples. For the United States, the survey firm Lucid sampled 2,352 adult respondents from the population of eligible voters. Lucid used quota sampling to produce a sample reflecting the US adult population with respect to gender, age, ethnicity, and geographic region. For each country, we screened out the 5% of respondents with the shortest completion times. For details on the number of respondents per country and their demographic characteristics, see Tables S1–S8.

### Sample weights

We used entropy balancing (45) to weight the sample to match the distribution of each country on gender, age, and education. Specifically, we matched on the % female in the population; on the % in three age categories (18–39, 40–59, and 60+); and on the % in three categories of highest educational attainment (% below upper secondary education, % with upper secondary or postsecondary nontertiary education, and % with tertiary education).

We calculated the population margins using the most recently available statistics from the Organisation for Economic Co-operation and Development (OECD). For age and gender, we used the OECD Population Statistics. For education, we used the table on share of population by educational attainment in the OECD Education at a Glance database. We dropped respondents for whom weights could not be constructed due to missing data on gender, age, or education, and we trimmed the weights at 6. The Online Supplementary Material provides both weighted and unweighted analyses.

### Experimental procedure

The survey began by obtaining consent and by screening out subjects who were not adult citizens of the country, were not eligible to vote in the country, or did not pass simple attention checks. After measuring pretreatment variables, we informed respondents about whether their country was currently a member of NATO and about what the NATO treaty requires. For example, subjects in the UK learned, “The United Kingdom is a member of NATO. The NATO treaty says that if any member of NATO is attacked, the other members will take all necessary actions, including the use of armed force, to defend their ally.” We provided this information because, in the event of a real attack, commentators would mention their country's membership in NATO and discuss what the treaty requires. We characterized one common view about obligations under the NATO treaty. Research in the United States suggests that slightly different ways of characterizing the obligations would produce similar effects (42).

Respondents then considered a hypothetical scenario involving a *Target* country that might join NATO: either Bosnia, Georgia, Finland, or Sweden. We told subjects, “There is much discussion about whether *Target* will become a member of NATO.” We then randomized whether subjects learned that the target joined NATO. Thus, half of the subjects read, “Suppose that *Target* becomes a member of NATO, and then Russia attacks *Target*.” The other half read, “Suppose that *Target* does not become a member of NATO, and then Russia attacks *Target*.” We asked all subjects, “In that situation, do you think *Sender* should or should not use military force to defend *Target*?”—where *Sender* was replaced by the respondent's own country. There were four answer options: definitely should, probably should, probably should not, and definitely should not.

We then presented a second scenario. To make the two vignettes as different as possible, subjects who had been told in the first scenario that the *Target* was either Bosnia or Georgia received a second scenario in which the *Target* was either Sweden or Finland, and vice versa. Moreover, subjects who were told in the first scenario that the *Target* joined NATO received a second scenario in which the *Target* did not join NATO, and vice versa. We then asked, “In that situation, do you think *Sender* should or should not use military force to defend *Target*?”

Thus, each subject evaluated two scenarios: one involving either Bosnia or Georgia and one involving either Finland or Sweden, and one in which the *Target* joined NATO and one in which it did not. The survey concluded by collecting additional demographic and attitudinal variables. The Online Supplementary Material provides the full text of the US version of the questionnaire as an example.

### Survey translations

We designed the questionnaire in English and professionally translated the questionnaire into each country's language(s).

### Variables

We constructed two measures of our dependent variable, support for military force. Our main measure, *Y_pct*, was 0 if the respondent selected “definitely should not” or “probably should not” and 100 if the respondent selected “probably should” or “definitely should.” Our secondary measure, *Y_scale*, was coded such that “definitely should not” = 0, “probably should not” = 33, “probably should” = 67, and “definitely should” = 100. Following our preregistration, the article presents findings based on *Y_pct*, but the Online Supplementary Material shows that our key conclusions held when we used *Y_scale*.

Our main predictor variable, *Member*, was coded as 1 if the target joined NATO and 0 if the target did not join NATO. We also constructed indicators for names of *Sender* and *Target* countries. Finally, we included an individual-level moderator variable, *NATO_good*, which measured whether the respondent agreed or disagreed that NATO membership is a good thing for their own country. *NATO_good* had five levels: agree strongly, agree, neither agree nor disagree, disagree, and disagree strongly.

For information on additional covariates employed in our analyses, see the supporting tables in the Online Supplementary Material.

### Analysis

We generated Fig. 2 by regressing *Y_pct* on a full set of interactions between *Sender*, *Target*, and *Member* (along with all lower-order terms) and then computing the average treatment effect—the effect of target membership in NATO—by giving equal weight to each combination of sender and target. In our supporting figures in the Online Supplementary Material, we also recreated this figure in the same manner but (i) using our alternative measure of the dependent variable, (ii) not employing the sample weights, and (iii) focusing on specific subsets of our sample as defined by key demographic characteristics.

We generated Fig. 3 by regressing *Y_pct* on a full set of interactions between *Sender*, *Target*, and *Member* (along with all lower-order terms) and then computing average support for using force to defend each non-NATO target by giving equal weight to each sender. In our supporting figures in the Online Supplementary Material, we also recreated this figure in the same manner but (i) using our alternative measure of the dependent variable and (ii) not employing the sample weights.

We generated Fig. 4 by regressing *Y_pct* on a full set of interactions between *Member*, *Sender*, and *NATO_good* and then computing the average treatment effect for each level of *NATO_good*, giving equal weight to each sender. In our supporting figures in the Online Supplementary Material, we also recreated this figure in the same manner but (i) using our alternative measure of the dependent variable and (ii) not employing the sample weights.

For all analyses, SE were clustered at the respondent level, and 95% normality-based CI were constructed.

### Preregistration

All hypotheses and analyses were preregistered at OSF (https://osf.io/pfzva/?view_only=ed65571a06904f3eb5ebab64608c9af0). The plan was posted on 2022 May 17, before fieldwork began.

## Supplementary Material

pgad206_Supplementary_Data

## Data Availability

Data and replication code are available on the Harvard Dataverse: https://doi.org/10.7910/DVN/SEGDGF.

## References

[1] Leeds BA . 2003. Alliance reliability in times of war: explaining state decisions to violate treaties. Int Organ. 57(4):801–827.

[2] Leeds BA, Long AG, Mitchell SM. 2000. Reevaluating alliance reliability. J Conflict Resolut. 44(5):686–699.

[3] Berkemeier M, Fuhrmann M. 2018. Reassessing the fulfillment of alliance commitments in war. Res Politics. 5(2):205316801877969.

[4] Lee S . 2023. The strong, the weak, and the honored: examining the decline in honored alliances post-1945. Int Interact. 49(1):114–131.

[5] Stimson JA, MacKuen MB, Erikson RS. 1995. Dynamic representation. Am Polit Sci Rev. 89(3):543–565.

[6] Burstein P . 2003. The impact of public opinion on public policy: a review and an agenda. Polit Res Q. 56(1):29–40.

[7] Tomz M, Weeks JLP, Yarhi-Milo K. 2020. Public opinion and decisions about military force in democracies. Int Organ. 71(1):119–143.

[8] Chu JA, Recchia S. 2022. Does public opinion affect the preferences of foreign policy leaders? Experimental evidence from the UK parliament. J Polit. 84(3):1874–1877.

[9] Lin-Greenberg E . 2021. Soldiers, pollsters, and international crises: public opinion and the military’s advice on the use of force. Foreign Policy Anal. 17(3):orab009.

[10] Baum MA, Potter PBK. 2015. War and democratic constraint: how the public influences foreign policy. Princeton (NJ): Princeton University Press.

[11] Gartzke E, Gleditsch K. 2004. Why democracies may actually be less reliable allies. Am J Polit Sci. 48(4):775–795.

[12] Chiba D, Johnson JC, Leeds BA. 2015. Careful commitments: democratic states and alliance design. J Polit. 77(4):968–982.

[13] Fjelstul JC, Reiter D. 2019. Explaining incompleteness and conditionality in alliance agreements. Int Interact. 45(6):976–1002.

[14] Kertzer JD . 2022. Re-assessing elite-public gaps in political behavior. Am J Pol Sci. 66(3):539–553.

[15] Tomz M, Weeks JLP. 2021. Military alliances and public support for war. Int Studi Q. 65(3):811–824.

[16] Guenther L, Musgrave P. 2022. New questions for an old alliance: NATO in cyberspace and American public opinion. J Glob Secur Stud. 7(4):ogac024.

[17] Chilton AS, Tingley DH. 2014. Why the study of international law needs experiments. Colum J Transnatl Law. 52(1):173–239.

[18] Kreps S, Maxey S. 2017. Mechanisms of morality: sources of support for humanitarian intervention. J Conflict Resolut. 62(8):1814–1842.

[19] Kertzer JD, Brutger R. 2016. Decomposing audience costs: bringing the audience back into audience cost theory. Am J Polit Sci. 60(1):234–249.

[20] Miller G . 2012. The shadow of the past: reputation and military alliances before the First World War. Ithaca: Cornell University Press.

[21] Gibler D . 2008. The costs of reneging: reputation and alliance formation. J Confl Resolut. 52(3):426–454.

[22] Crescenzi MJC, Kathman JD, Kleinberg KB, Wood RM. 2012. Reliability, reputation, and alliance formation. Int Stud Q. 56:259–274.

[23] Mattes M . 2012. Reputation, symmetry, and alliance design. Int Organ. 66(4):679–707.

[24] Johnson JC . 2015. The cost of security: foreign policy concessions and military alliances. J Peace Res. 52(5):665–679.

[25] Johnson JC . 2016. Alliance treaty obligations and war intervention. Confl Manag Peace Sci. 33(5):451–468.

[26] Tomz M, Weeks JLP. 2013. Public opinion and the democratic peace. Am Polit Sci Rev. 107(3):849–865.

[27] Kertzer JD, Powers KE, Rathbun BC, Iyer R. 2014. Moral support: how moral values shape foreign policy attitudes. J Polit. 76(3):825–840.

[28] Reifler J, et al 2014. Prudence, principle and minimal heuristics: British public opinion towards the use of military force in Afghanistan and Libya. Br J Polit Int Relat. 16(1):28–55.

[29] Kreps SE, Wallace GP. 2016. International law, military effectiveness, and public support for drone strikes. J Peace Res. 53(6):830–844.

[30] Bansak K, Hainmueller J, Hangartner D. 2017. Europeans support a proportional allocation of asylum seekers. Nat Hum Behav. 1(7):0133.

[31] Bansak K . 2020. Comparative causal mediation and relaxing the assumption of no mediator-outcome confounding: an application to international law and audience costs. Polit Anal. 28(2):222–243.

[32] Alley J . 2022. Elite cues and public attitudes towards military alliances. J Confl Resolut. doi: 10.1177/00220027221143963

[33] Lau RR, Redlawsk DP. 2001. Advantages and disadvantages of cognitive heuristics in political decision making. Am J Polit Sci. 45(4):951–971.

[34] Boudreau C . 2022. Heuristics and cues. Handbook on Politics and Public Opinion. 272–282.

[35] Leeds BA, Mattes M, Vogel J. 2009. Interests, institutions, and the reliability of international commitments. Am J Polit Sci. 53(2):461–476.

[36] Martin LL . Against compliance. 2012. In: Dunoff JL, Pollack MA, editors. Interdisciplinary perspectives on international law and international relations. New York: Cambridge University Press. p. 591–610.

[37] Chu JA, Ko J, Liu A. 2021. Commanding support: values and interests in the rhetoric of alliance politics. Int Interact. 47(3):477–503.

[38] Leeds BA . 2003. Do alliances deter aggression? The influence of military alliances on the initiation of militarized interstate disputes. Am J Polit Sci. 47(3):427–439.

[39] Clare J . 2013. The deterrent value of democratic allies. Int Stud Q. 57(3):545–555.

[40] Johnson JC, Wolford S. 2022. Alliance reliability and dispute escalation. J Confl Resolut. 67(4):617–641.

[41] Kucharski L . 2019. Russian multi-domain strategy against NATO: information confrontation and U.S. forward-deployed nuclear weapons in Europe. No. LLNL-TR-767588: Livermore (CA): Lawrence Livermore National Lab.

[42] Reiter D, Greenhill B. N.d. Manipulating public beliefs about alliance compliance: a survey experiment. Working Paper

[43] Blankenship B . 2020. Promises under pressure: statements of reassurance in U.S. alliances. Int Stud Q. 64(4):1017–1030.

[44] Kim T . 2011. Why alliances entangle but seldom entrap states. Secur Stud. 20(3):350–377.

[45] Hainmueller J . 2012. Entropy balancing for causal effects: a multivariate reweighting method to produce balanced samples in observational studies. Polit Analysis. 20(1):25–46.

