# Correction to: How membership in the North Atlantic Treaty Organization transforms public support for war

**DOI:** 10.1093/pnasnexus/pgad381

**Published:** 2023-11-15

This is a correction to: Michael Tomz, Jessica L P Weeks, Kirk Bansak, How membership in the North Atlantic Treaty Organization transforms public support for war, *PNAS Nexus*, Volume 2, Issue 7, July 2023, pgad206, https://doi.org/10.1093/pnasnexus/pgad206

During a retroactive audit conducted by *PNAS Nexus*, it was discovered that this paper was missing a statement acknowledging compliance with the *PNAS Nexus* Human and Animal Participants and Clinical Trials policy:

This research was approved by Stanford University's Institutional Review Board (protocol ID 34881). An information sheet presented to respondents at the start of the survey was used to inform participants about the purpose of the study and their rights, and to obtain their consent to participate.

This error has been corrected in the original article.

