# Supplementary material for: How membership in the North Atlantic Treaty Organization transforms public support for war
Source: PNAS Nexus. 2023 Jul 5;2(7):pgad206. doi: 10.1093/pnasnexus/pgad206 (PMC10321487; doi:10.1093/pnasnexus/pgad206)
Supplement: pgad206_Supplementary_Data [file pgad206_supplementary_data.pdf]

Supporting Information for

How Membership in the North Atlantic Treaty Organization Transforms Public Support for War

Michael Tomz, Jessica L. P. Weeks, and Kirk Bansak

Corresponding author: Kirk Bansak
Email: kbansak@berkeley.edu

This PDF file includes:

Supporting Text
Figures S1 to S21
Tables S1 to S8
Survey Questionnaire

SUPPORTING INFORMATION (SI) FOR:
HOW NATO MEMBERSHIP TRANSFORMS PUBLIC SUPPORT FOR WAR

TABLE OF CONTENTS

I. SUPPORTING TEXT 4
Characterizing Bosnia and Georgia versus Finland and Sweden 4
(1) Bosnia and Georgia are less democratic 4
(2) The costs would be higher for defending Bosnia and Georgia 4
(3) The consequences for current NATO members of an attack would be lower if the target were Bosnia or Georgia 5
Influence of Attitudes about NATO Membership 5
Results in Non-NATO Countries 6
II. SUPPORTING FIGURES 8
All Respondents in NATO Sender Countries 8
Figure S1: Effect of Target Joining NATO, Overall and by Sender Country 8
Figure S2: Effect of Target Joining NATO, by Target Country 9
Figure S3: Effect of Target Joining NATO, by Sender Country when Target is Bosnia 10
Figure S4: Effect of Target Joining NATO, by Sender Country when Target is Georgia ... 11
Figure S5: Effect of Target Joining NATO, by Sender Country when Target is Finland 12
Figure S6: Effect of Target Joining NATO, by Sender Country when Target is Sweden 13
Figure S7: Effect of Target Joining NATO, by Attitudes about NATO Membership 14
Subgroups of Respondents in NATO Sender Countries 15
Figure S8: Respondents with “Left” Ideology 15
Figure S9: Respondents with “Center” Ideology 16
Figure S10: Respondents with “Right” Ideology 17
Figure S11: Female Respondents 18
Figure S12: Male Respondents 19
Figure S13: Democrats in the United States 20
Figure S14: Independents in the United States 21
Figure S15: Republicans in the United States 22
Figure S16: Respondents with Tertiary Education 23
Figure S17: Respondents with Above-Median Income 24
Figure S18: Respondents with High Political Interest 25
Figure S19: Respondents At Least 40 Years Old 26
Respondents in Non-NATO Sender Countries 27
Figure S20: Effect of Target Joining NATO 27
Figure S21: Effect of Target Joining NATO, By Attitudes about Joining NATO 28
III. SUPPORTING TABLES 29
Summary Statistics for Respondents in NATO Member Countries 29
Table S1: Number of Respondents in Each of the 13 NATO Member Countries 29
Table S2: Gender and Age of Sample in the 13 NATO Member Countries 29
Table S3: Education of Sample in the 13 NATO Member Countries 30
Table S4: Attitudes about NATO Membership in the 13 NATO Member Countries 30
Summary Statistics for Respondents in Non-NATO Member Countries 31

Table S5: Number of Respondents in Each of the 3 Non-NATO Member Countries 31
Table S6: Gender and Age of Sample in the 3 Non-NATO Member Countries 31
Table S7: Education of Sample in the 3 Non-NATO Member Countries 31
Table S8: Attitudes about NATO Membership in the 3 Non-NATO Member Countries.... 32
IV. QUESTIONNAIRE (UNITED STATES)... 33
Consent 33
Screening Questions.. 34
Pretreatment Questions 36
Randomized Treatments 41
Text of the Experiment 42
Additional Background Questions.. 47

I. SUPPORTING TEXT

Characterizing Bosnia and Georgia versus Finland and Sweden

As we explain in the main text, we predicted that joining NATO would have a stronger effect on public opinion when the target is Bosnia and Georgia relative to Finland and Sweden. We based this prediction on the expectation that, without a NATO commitment, support for defending Bosnia and would be lower than support for defending Finland or Sweden, due to three factors: (1) Bosnia and Georgia are perceived as less democratic than Finland and Sweden; (2) the costs of defending Bosnia and Georgia would likely be higher than the costs of defending Finland and Sweden, due to differences in military power, geographic location, and compatibility with NATO's force structure; and (3) for current NATO members, the economic and security consequences of a Russian attack would be lower if the target were Bosnia or Georgia than if the target were Finland or Sweden. Here we provide evidence corroborating these claims.

(1) Bosnia and Georgia are less democratic

Freedom House, a non-profit that conducts research and advocacy on democracy, annually rates the levels of access to political rights and civil liberties in countries around the world. According to Freedom House's 2022 Global Freedom Scores,[1] Sweden and Finland both received scores of 100 (out of 100), which denotes the highest level of freedom and corresponds to the status of "Free." In contrast, Bosnia received a score of 53 ("Partly Free") and Georgia a score of 58 ("Partly Free"), and both countries were further classified as being a "Transitional or Hybrid Regime" rather than a "Consolidated Democracy."

(2) The costs would be higher for defending Bosnia and Georgia

We base this claim on three factors: estimates of military power, estimates of economic power, and level of existing military coordination with NATO members.

In terms of current military power, Finland and Sweden have spent much more on their militaries over the past decade than Bosnia and Georgia. According to estimates by the Stockholm International Peace Research Institute (SIPRI), an independent international institute that researches global armament and maintains an annual military expenditure database,[2] Finland's and Sweden's military expenditures over 2012-2021 (in 2020 USD, in millions) totaled 39,713 and 61,400, respectively. In contrast, Bosnia's and Georgia's military expenditures over the same period were 1,759 and 3,520, respectively.

In terms of economic power, Sweden and Finland are much wealthier than Bosnia and Georgia. The World Bank reports that in 2022 US dollars (in millions), the GDPs of Finland and Sweden in 2022 were 299,155.24 and 627,437.90, respectively. In contrast, the GDPs of Bosnia and Georgia were 22,571.51 and 18,700.24, respectively.

Finally, Sweden and Finland have higher levels of military coordination with most current NATO members, compared to Bosnia and Georgia. Sweden and Finland are both part of the European Union (EU), which maintains a Common Security and Defence Policy (CSDP). As a result, most NATO members maintain regular military coordination and foreign policy alignment with Finland and Sweden. In contrast, Bosnia and Georgia are not part of the EU, and thus do not share the same level of defense or foreign policy alignment (though note that all four countries are part of various NATO coordination initiatives).[3]

---

[1] https://freedomhouse.org/countries/freedom-world/scores
[2] https://www.sipri.org/databases/milex
[3] https://www.nato.int/cps/em/natohq/topics_132726.htm

(3) The consequences for current NATO members of an attack would be lower if the target were Bosnia or Georgia

Finally, for current NATO members, the stakes of a Russian invasion of Bosnia or Georgia would be lower than the stakes of a Russian invasion of Finland or Sweden. While space does not permit an exhaustive analysis of the stakes, we note two important points.

First, the stakes are lower when the target is Bosnia or Georgia because the NATO countries in our sample tend to be much less economically integrated with Bosnia or Georgia than with Finland or Sweden. Finland and Sweden are members of the EU, making them part of a free-trade zone with most of the NATO countries we sampled (the three exceptions are Norway, the UK, and the US), fostering deep economic integration. Thus, an attack on Finland and Sweden could have significant economic consequences for most of the NATO countries in our sample. In contrast, Bosnia and Georgia do not have nearly the same depth of economic ties with the NATO countries in our sample and an attack on them would result in less economic spillover to those countries.

Second, a Russian attack on Finland or Sweden could have particularly dire social and political consequences for NATO countries due to the free movement of people within the EU. As noted, Finland, Sweden, and most of the NATO countries in our sample share EU membership. EU membership permits individuals to move about freely within the EU for any reason they wish, which means that a Russian attack on Finland or Sweden could produce an influx of people to many of the countries in our NATO sample. This in turn could produce significant social and political dislocation. The movement of people between NATO countries and Bosnia and Georgia, in contrast, is currently much more restricted (Bosnia and Georgia also have smaller populations than Finland or Sweden). Thus, a Russian attack on Bosnia or Georgia would not produce comparable flows of individuals into, and hence destabilization of, the NATO countries we studied.

Influence of Attitudes about NATO Membership

As described in the main text, we hypothesized that alliances should more sharply increase support for war among voters who *a priori* prize the alliance than among those who doubt its value. We tested this by analyzing the heterogeneity of the NATO treatment effect across subsets of respondents characterized by the extent to which they agreed (or disagreed) that their own country's membership in NATO is a good thing, and the results (shown in Figure 4) provided strong support for our hypothesis: the effect of the NATO treatment on support for war was highest among voters with the strongest pro-NATO attitudes (i.e. those who agreed strongly), and that effect consistently declined with both statistical and substantive significance as enthusiasm for NATO waned.

Here, we further investigate the precise relationship between NATO attitudes and the NATO treatment effect in supplementary analyses that were not pre-registered. (For these analyses, as with the analyses reported in the main text, we employ our sample weights and cluster standard errors at the respondent level.) Specifically, we distinguish between treatment effect heterogeneity and causal moderation (*42*). In our context, treatment effect heterogeneity refers to the simple differences or heterogeneity of the NATO treatment effect across respondents with different attitudes toward NATO, which is precisely what is uncovered in our main analysis highlighted in Figure 4. In contrast, causal moderation refers to the possibility that those differences in the NATO treatment effect can be causally attributed to NATO attitudes *per se*, rather than being driven by other potential influences that happen to be correlated with NATO attitudes.

To facilitate a smoother interpretation and comparison between treatment effect heterogeneity and causal moderation, we first coarsened our measurement of NATO attitudes. Specifically, we dichotomized

voters' attitudes toward NATO into a pro-NATO indicator, which took a value of 1 for voters who agreed or strongly agreed that their own country's membership in NATO is a good thing, and a value of 0 otherwise. We then estimated the treatment effect heterogeneity by following the same analysis used to produce Figure 4, but using the dichotomized pro-NATO indicator in place of the original 5-point measurement. Giving equal weight to all countries, we find that having pro-NATO attitudes (relative to not) is associated with an increase in the NATO treatment effect of 18.4 percentage points (95% CI: [15.7 , 21.0]). In other words, while there is a strong surge in support for defending a target country when that target has joined NATO, that surge is much larger among voters who have pro-NATO attitudes than among voters who do not. We also find that this positive increase in the NATO treatment effect holds for all 13 of the sender countries individually, with statistical significance at the 95% level for 12 out of the 13.

The ensuing question is then whether that increase in the NATO treatment effect is not only associated with but also causally attributable to pro-NATO attitudes. This moves us from treatment effect heterogeneity to causal moderation. To assess this, we employed the causal moderation estimation framework from (*42*) to estimate the average treatment moderation effect (ATME), which corresponds to the causal influence of pro-NATO attitudes on the effect of the NATO treatment. Specifically, we applied the parallel-regression approach to estimate the ATME by appropriately controlling for a number of respondent characteristics that are upstream of pro-NATO attitudes and could be responsible for the treatment effect heterogeneity we observed (i.e. characteristics that could have an influence on both pro-NATO attitudes and responsiveness to the treatment). These characteristics include gender, age, education, income, political ideology, employment status, whether the respondent was born in their country, degree of political interest, level on a nationalism index, and level on a cosmopolitanism index. We estimated the ATME separately for each country and then computed an average of the estimates giving equal weight across all countries, thereby providing a result that is directly comparable to the 18.4 percentage-point difference in effects we computed above for treatment effect heterogeneity. Our average estimate of the ATME is 15.7 percentage points (95% CI: [13.0 , 18.3]). The country-specific estimates of the ATME are positive again for all 13 countries, and statistically significant at the 95% level for 10 out of the 13.

In sum, the causal moderation analysis provides results that, while slightly smaller in magnitude, are largely similar to the results of the treatment effect heterogeneity analysis. Of course, we do not believe we have necessarily controlled for every possible relevant variable in the causal moderation analysis, and such a feat would be practically impossible. Nonetheless, that our estimates remained robust (with very little substantive change) after adjusting for a broad range of variables of high theoretical salience provides compelling (even if tentative) evidence that NATO attitudes do indeed directly influence the effect of NATO on public opinion.

Results in Non-NATO Countries

In addition to thirteen NATO countries, we also fielded our experiment in three non-NATO countries: Austria, Sweden, and Switzerland. (Note that Sweden was not allowed as the target country for respondents in Sweden.) The purpose of fielding the experiment in these countries was to undertake additional, complementary analyses alongside the primary tests we describe in the main text.

Specifically, we tested three preregistered predictions. First, we hypothesized that the effect of the target joining NATO would be smaller among subjects in the three non-NATO countries than among subjects in NATO countries, because the target joining NATO only generates an obligation for fellow NATO members. Second, we predicted that the effect of the target joining NATO would be larger among subjects in Sweden than among respondents in Austria or Switzerland. We expected this because Sweden was considering joining NATO at the time of the survey and voters might have been answering our

questions in anticipation of a future bid for NATO accession. Third, following similar reasoning, we expected the effect of the target joining NATO to be larger for subjects who said that their country joining NATO would be a good thing than among subjects who did not. Figures A20 and A21 show that all three of these hypotheses were supported.

II. SUPPORTING FIGURES

All Respondents in NATO Sender Countries

Figure S1: Effect of Target Joining NATO, Overall and by Sender Country

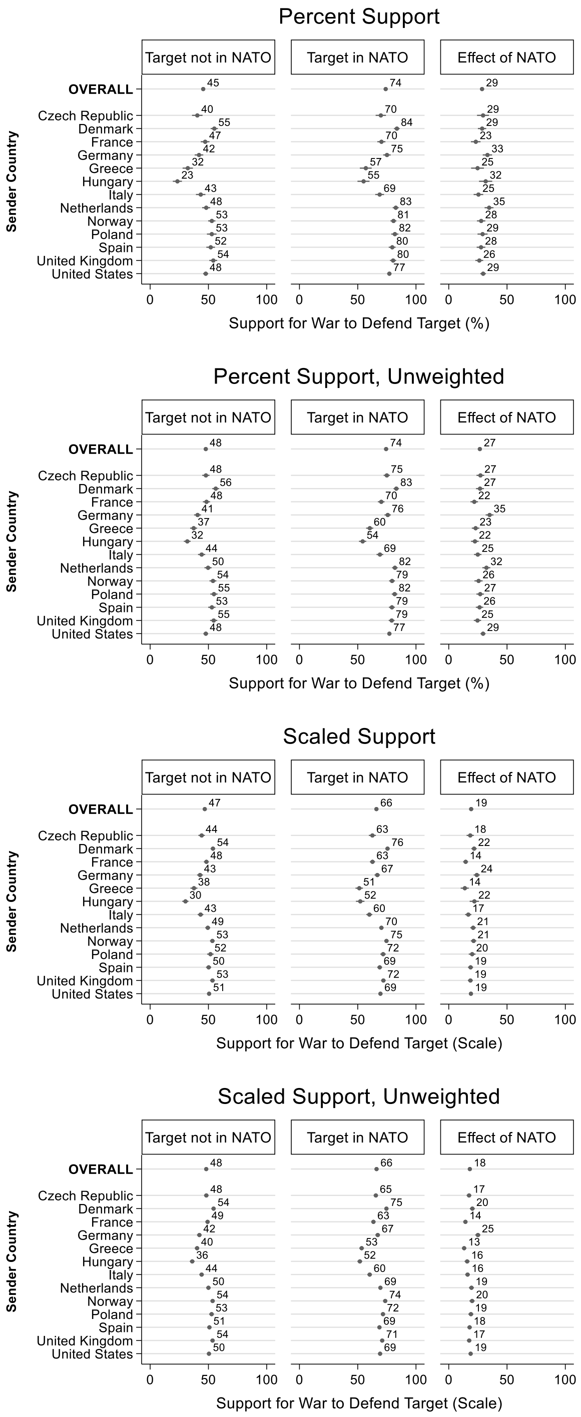

Note: The figure gives equal weight to each target country. Overall was estimated by giving equal weight to each sender country.

Figure S2: Effect of Target Joining NATO, by Target Country

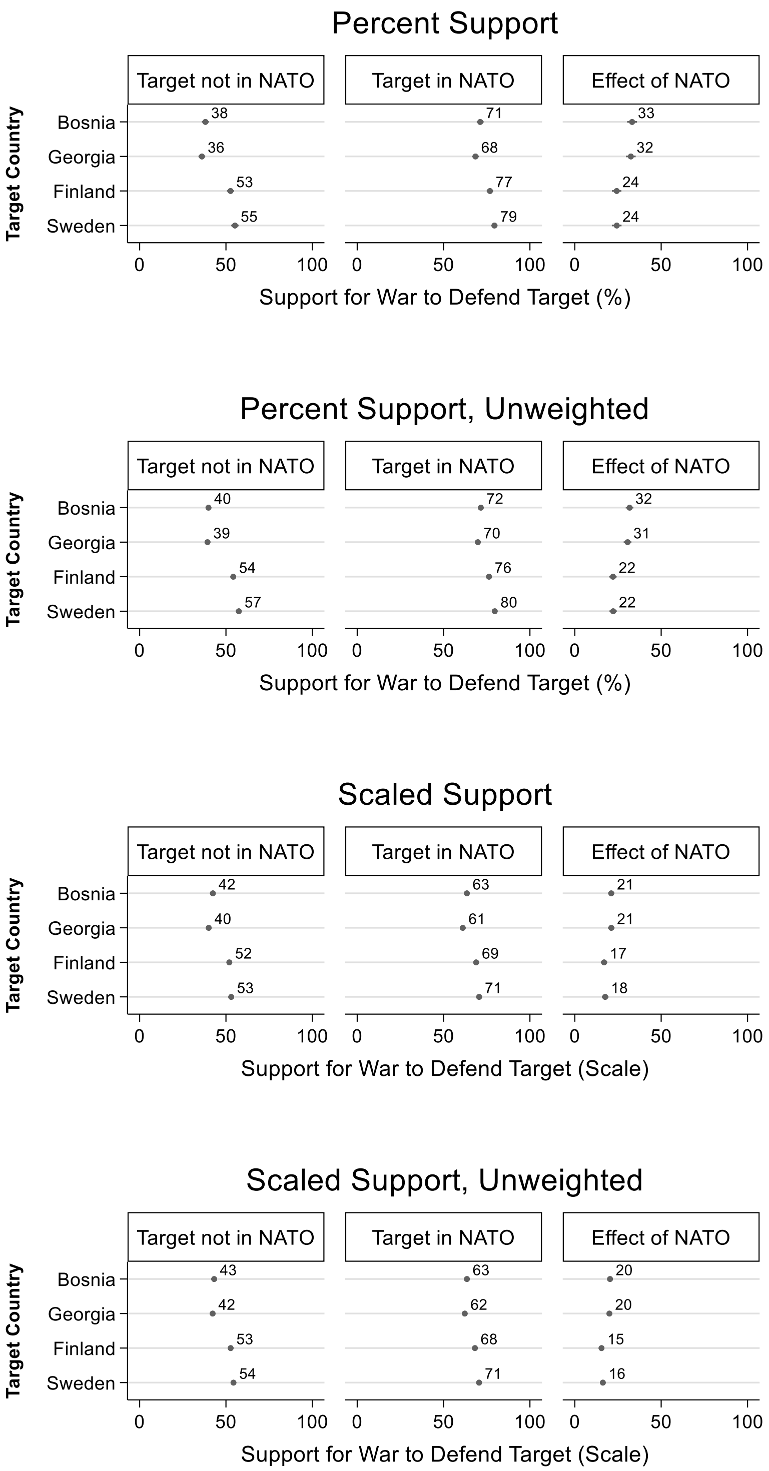

Note: The figure gives equal weight to each sender country.

Figure S3: Effect of Target Joining NATO, by Sender Country when Target is Bosnia

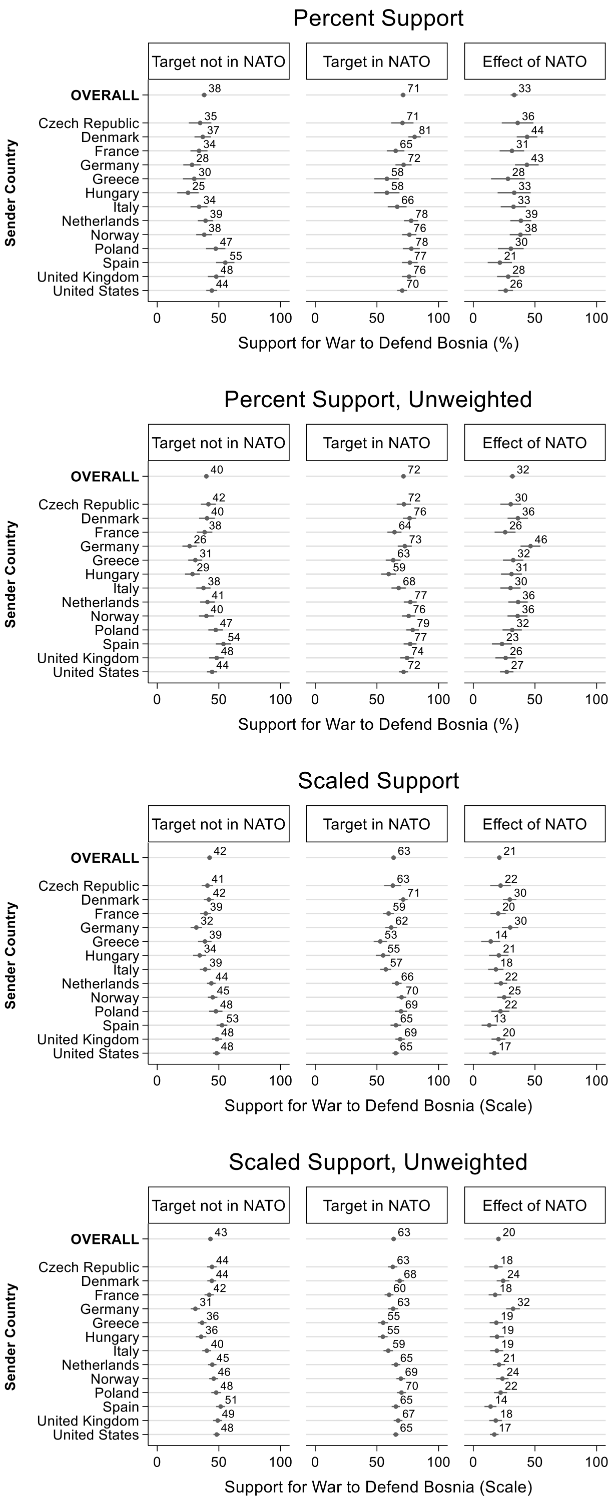

Note: Overall was estimated by giving equal weight to each sender country.

Figure S4: Effect of Target Joining NATO, by Sender Country when Target is Georgia

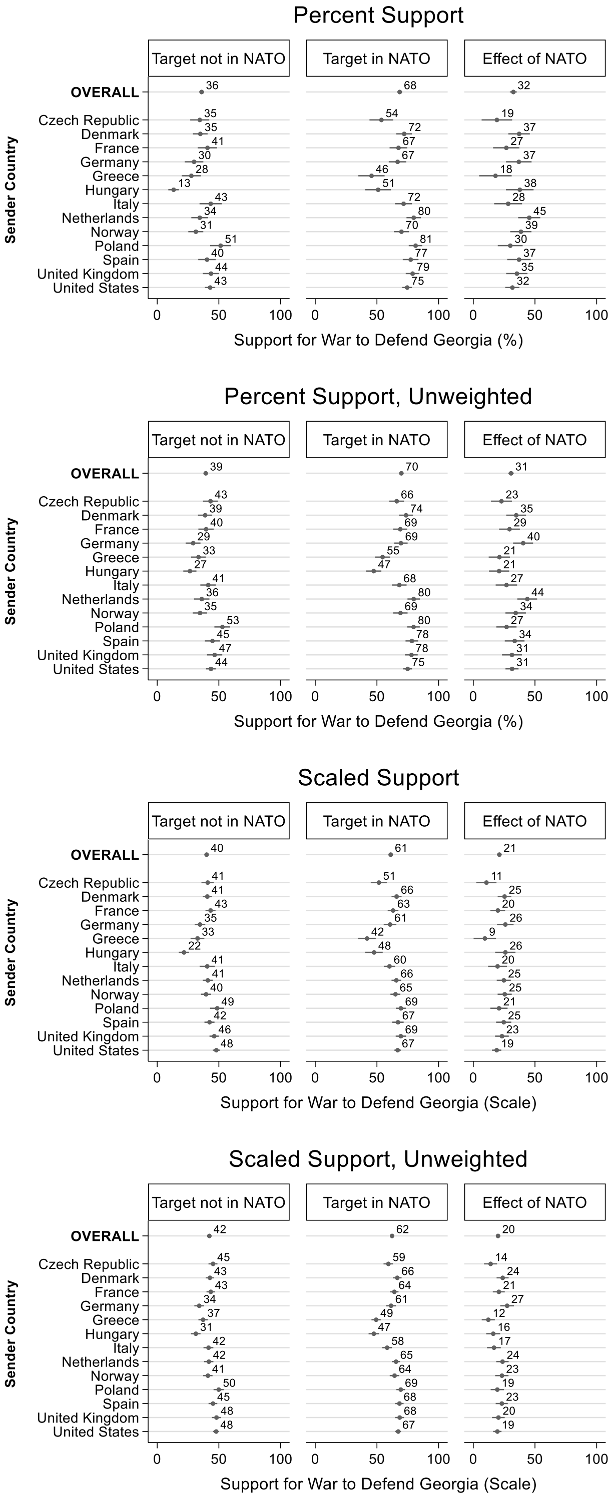

Note: Overall was estimated by giving equal weight to each sender country.

Figure S5: Effect of Target Joining NATO, by Sender Country when Target is Finland

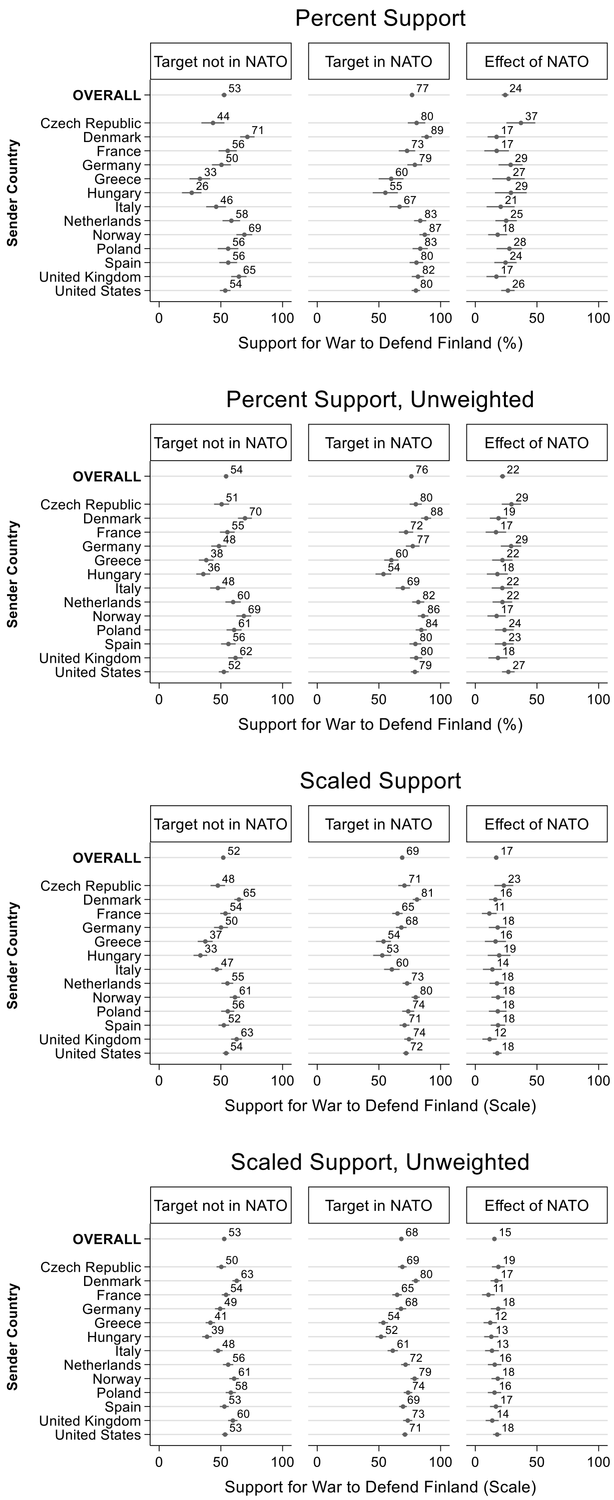

Note: Overall was estimated by giving equal weight to each sender country.

Figure S6: Effect of Target Joining NATO, by Sender Country when Target is Sweden

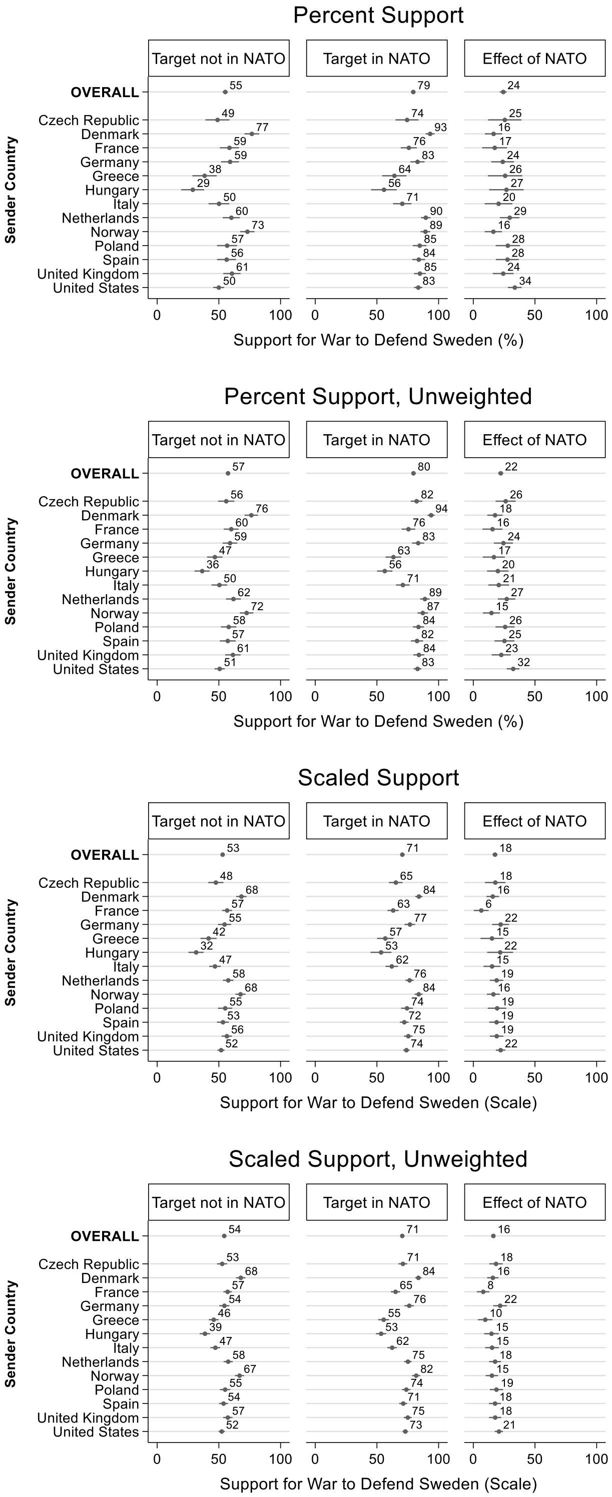

Note: Overall was estimated by giving equal weight to each sender country.

Figure S7: Effect of Target Joining NATO, by Attitudes about NATO Membership

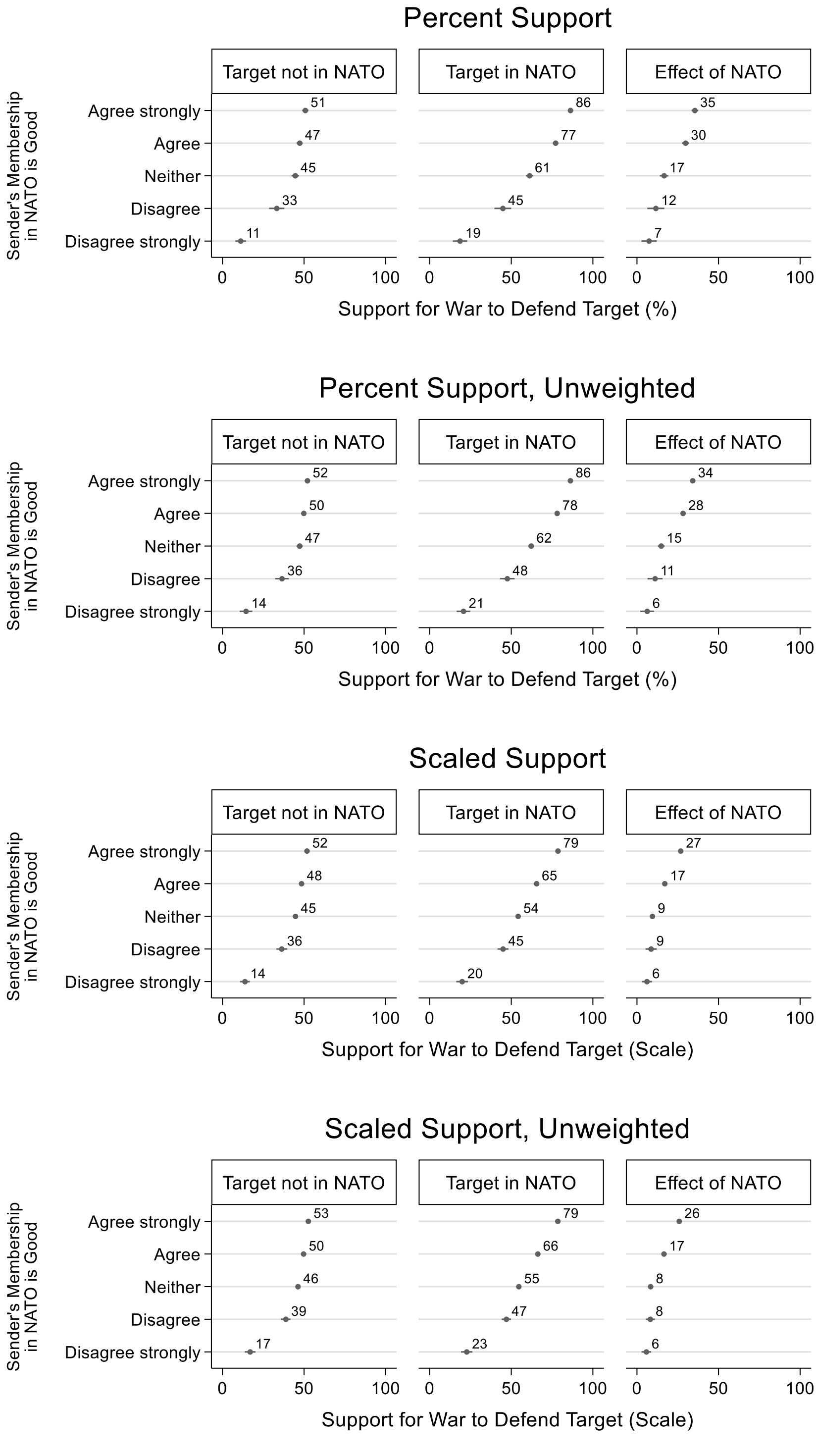

Note: The figure gives equal weight to each sender country.

Subgroups of Respondents in NATO Sender Countries

Figure S8: Respondents with "Left" Ideology

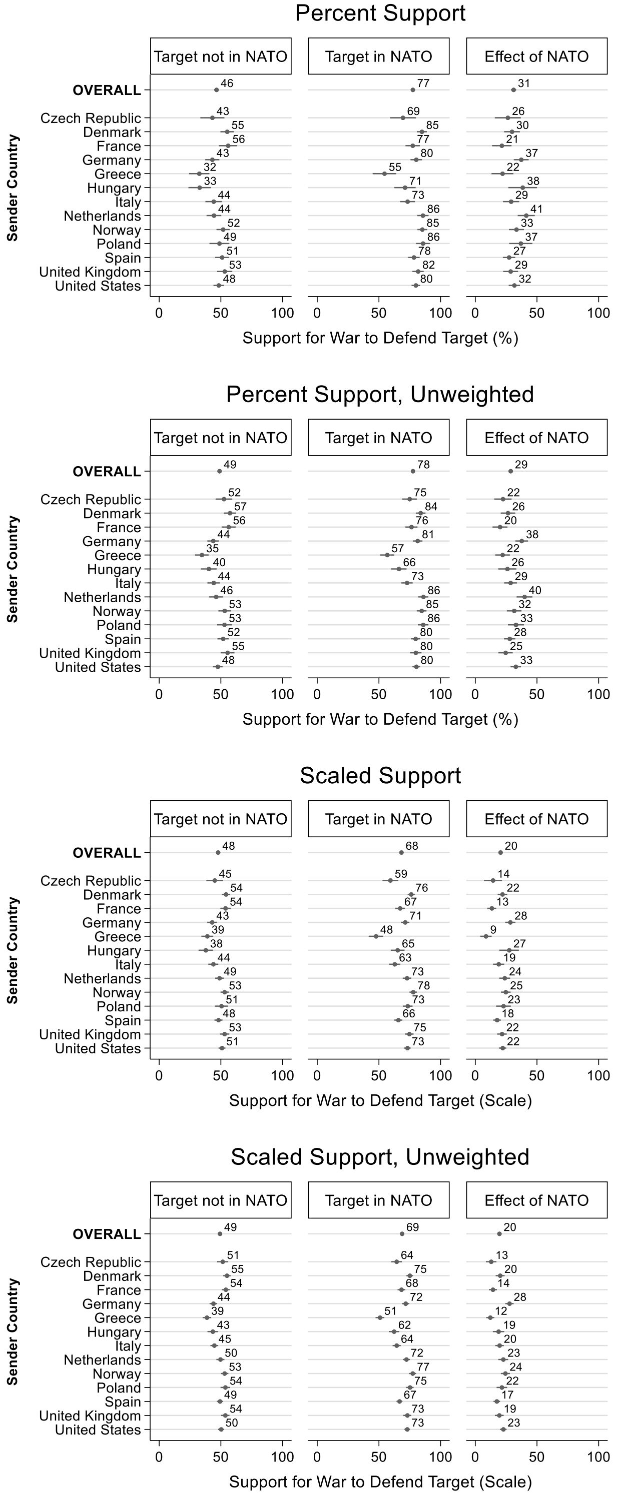

Note: The figure gives equal weight to each target country. Overall was estimated by giving equal weight to each sender country.

Figure S9: Respondents with “Center” Ideology

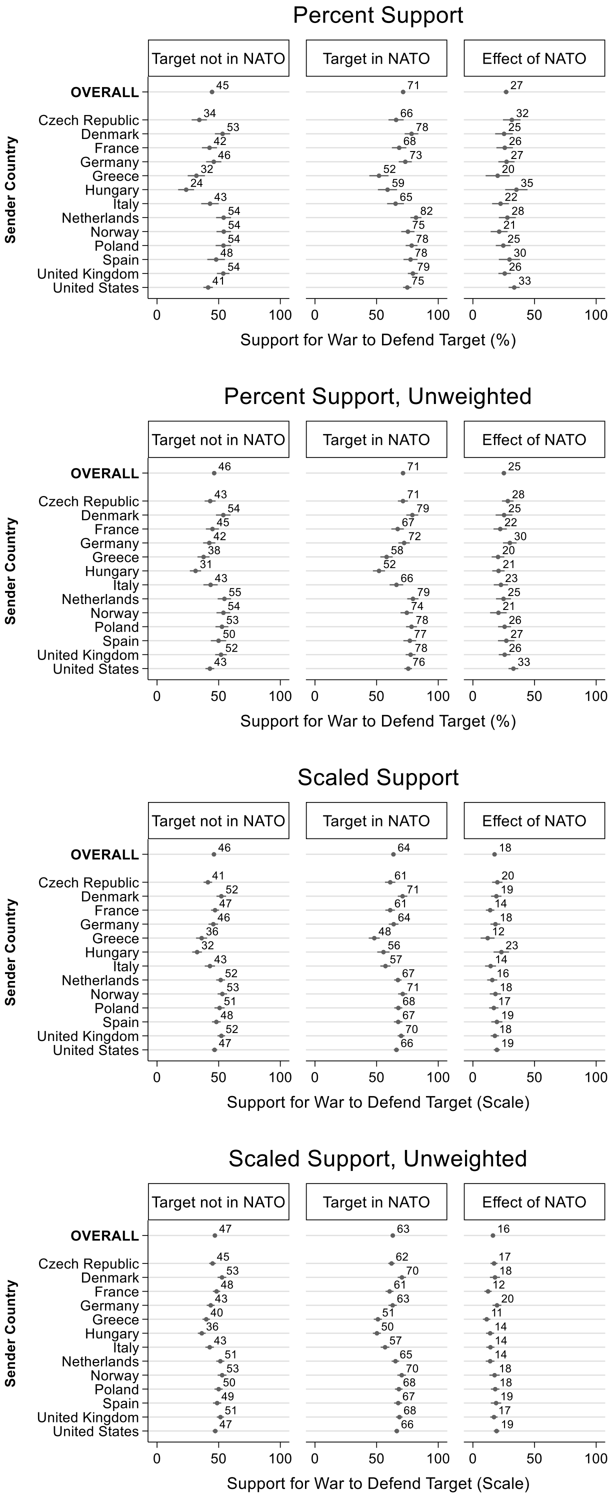

Note: The figure gives equal weight to each target country. Overall was estimated by giving equal weight to each sender country.

Figure S10: Respondents with "Right" Ideology

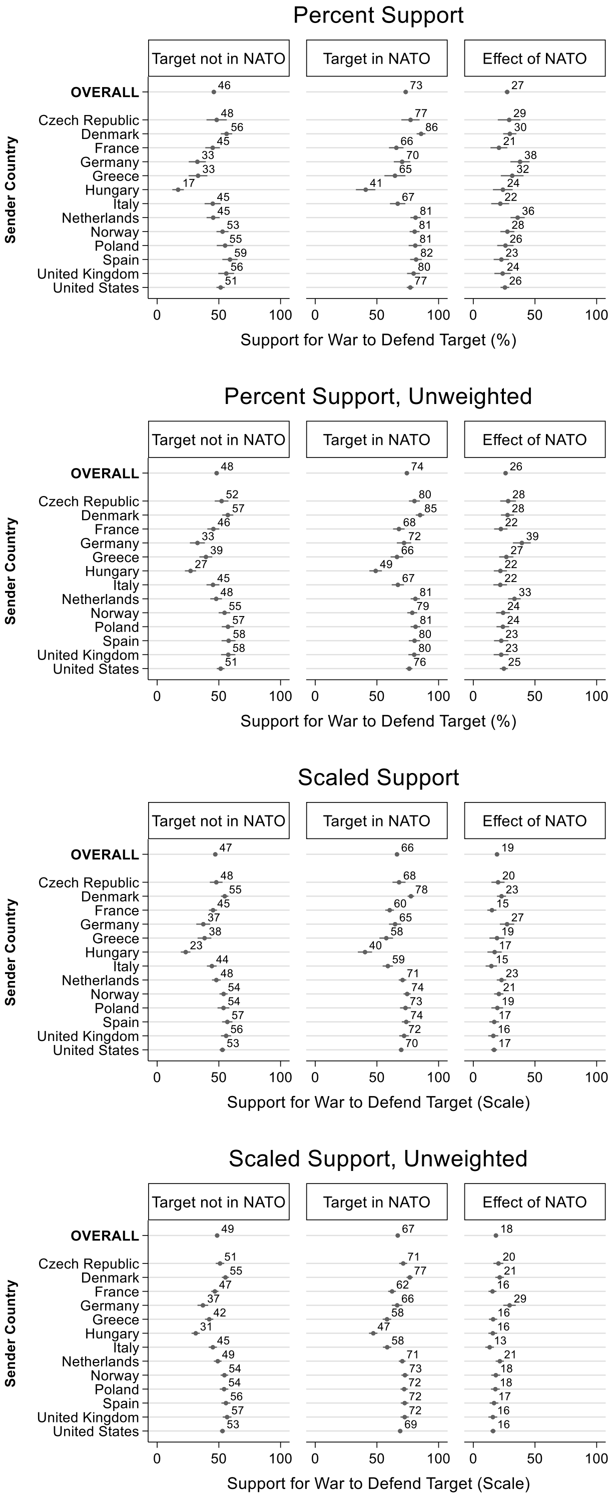

Note: The figure gives equal weight to each target country. Overall was estimated by giving equal weight to each sender country.

Figure S11: Female Respondents

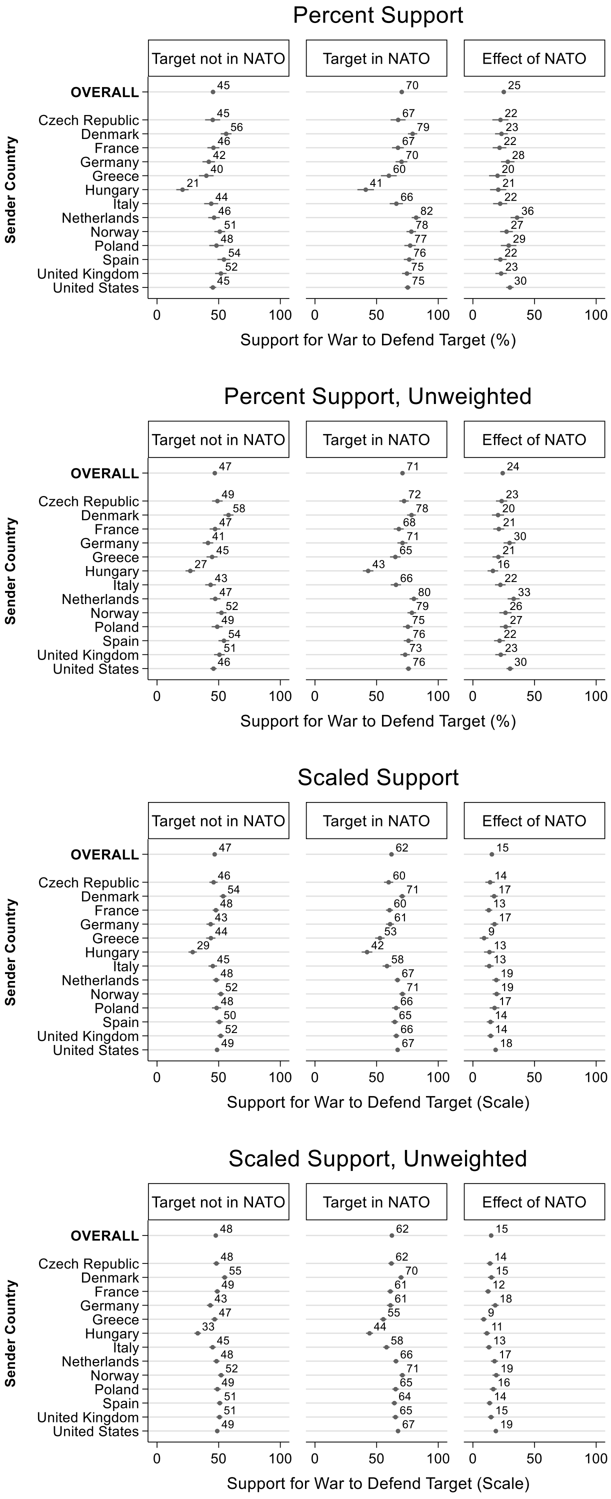

Note: The figure gives equal weight to each target country. Overall was estimated by giving equal weight to each sender country.

Figure S12: Male Respondents

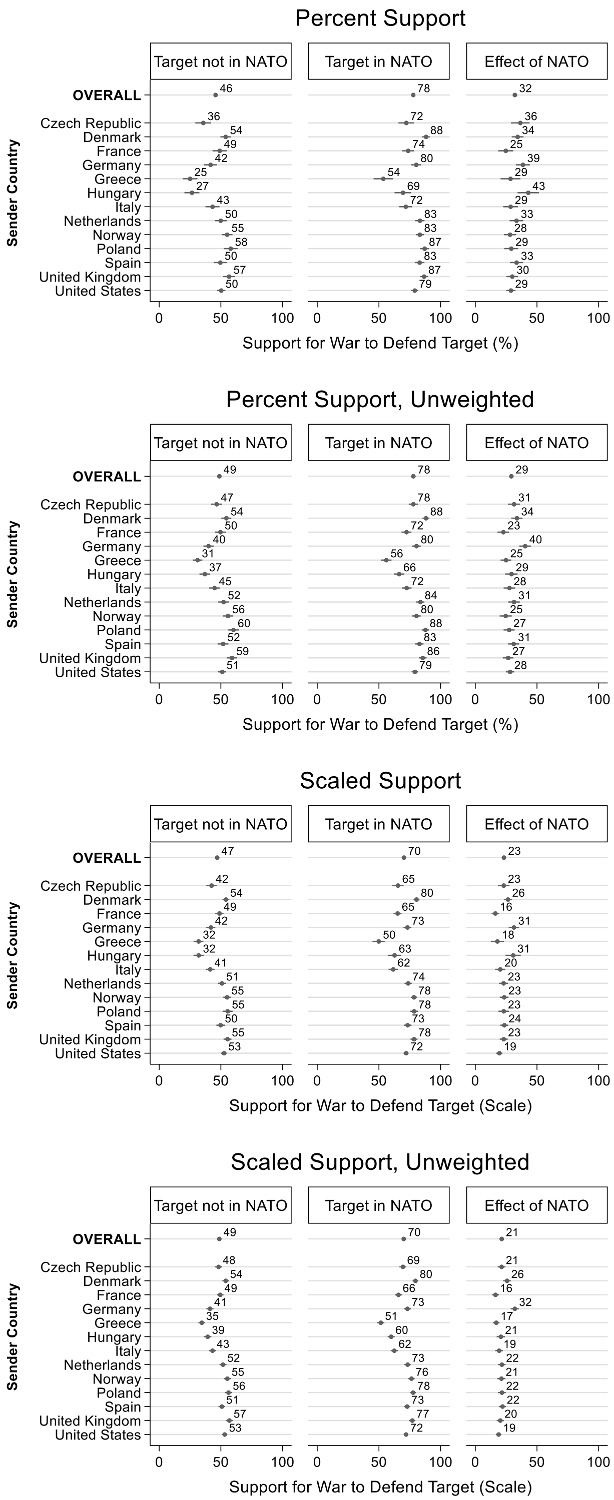

Note: The figure gives equal weight to each target country. Overall was estimated by giving equal weight to each sender country.

Figure S13: Democrats in the United States

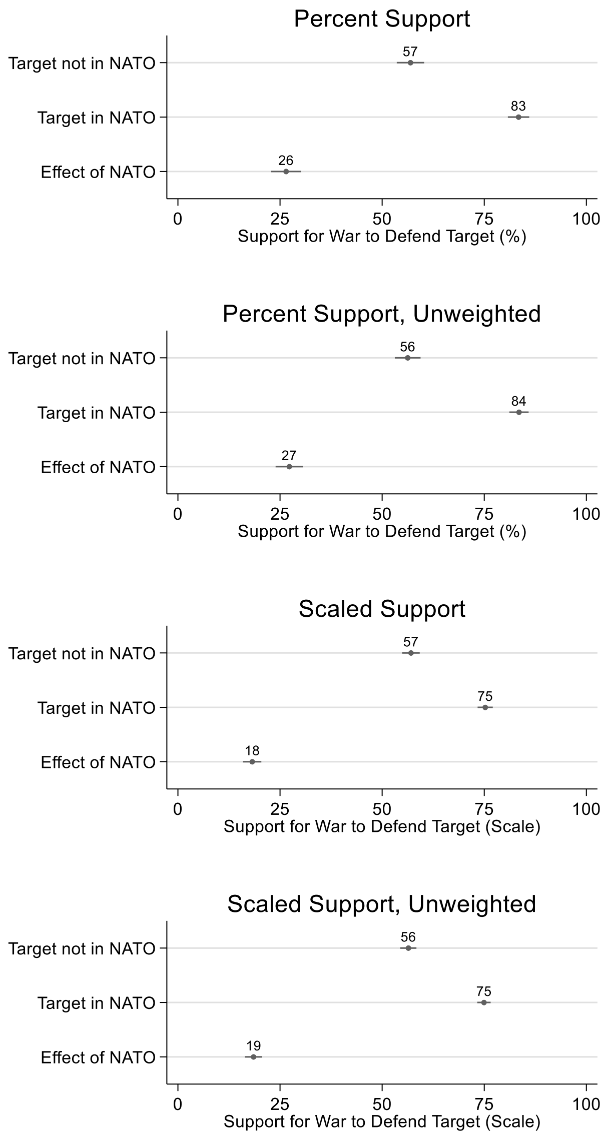

Note: The figure gives equal weight to each target country.

Figure S14: Independents in the United States

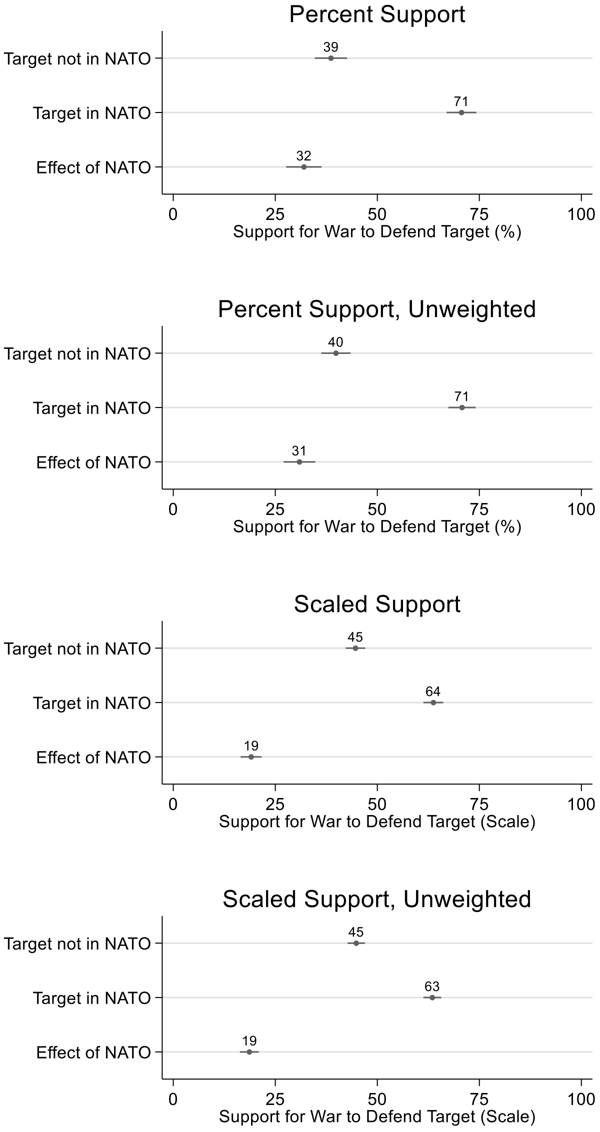

Note: The figure gives equal weight to each target country.

Figure S15: Republicans in the United States

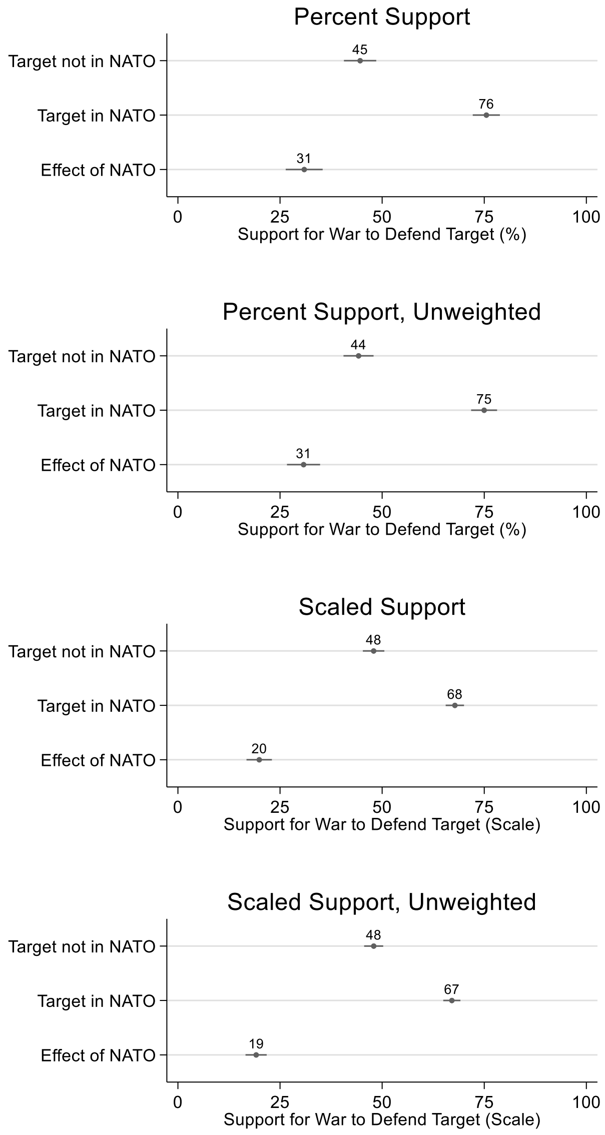

Note: The figure gives equal weight to each target country.

Figure S16: Respondents with Tertiary Education

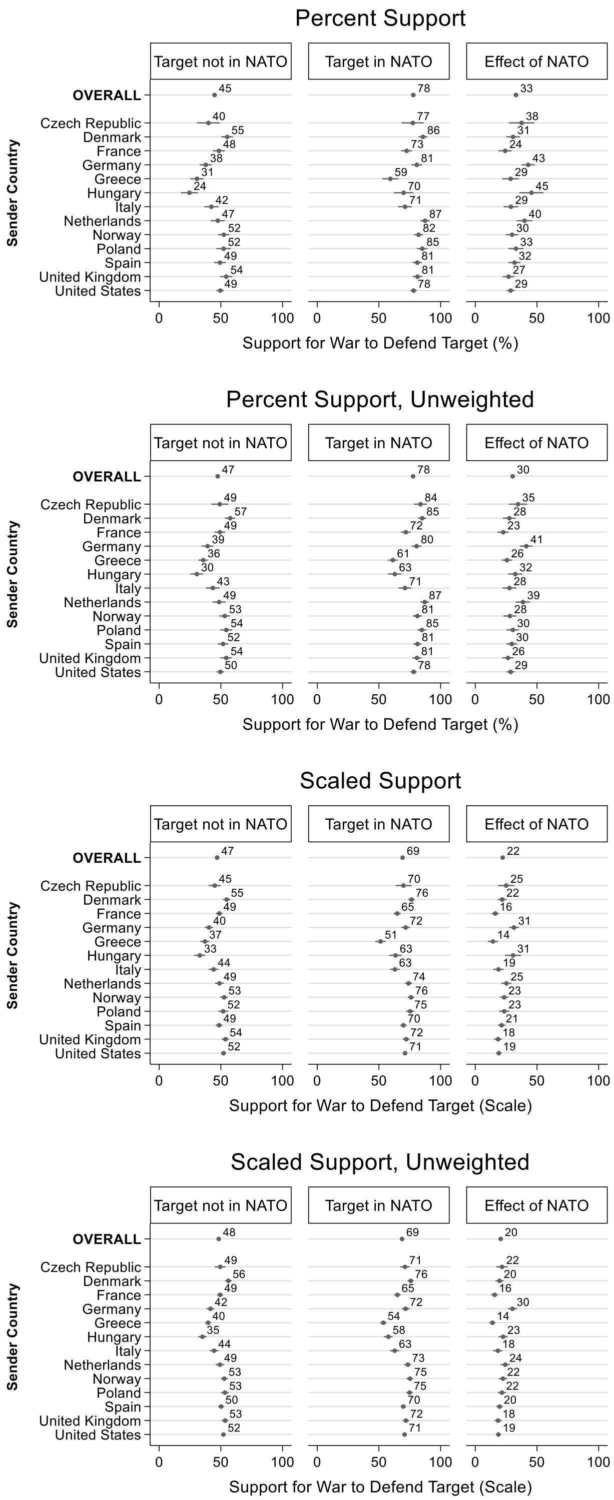

Note: The figure gives equal weight to each target country. Overall was estimated by giving equal weight to each sender country.

Figure S17: Respondents with Above-Median Income

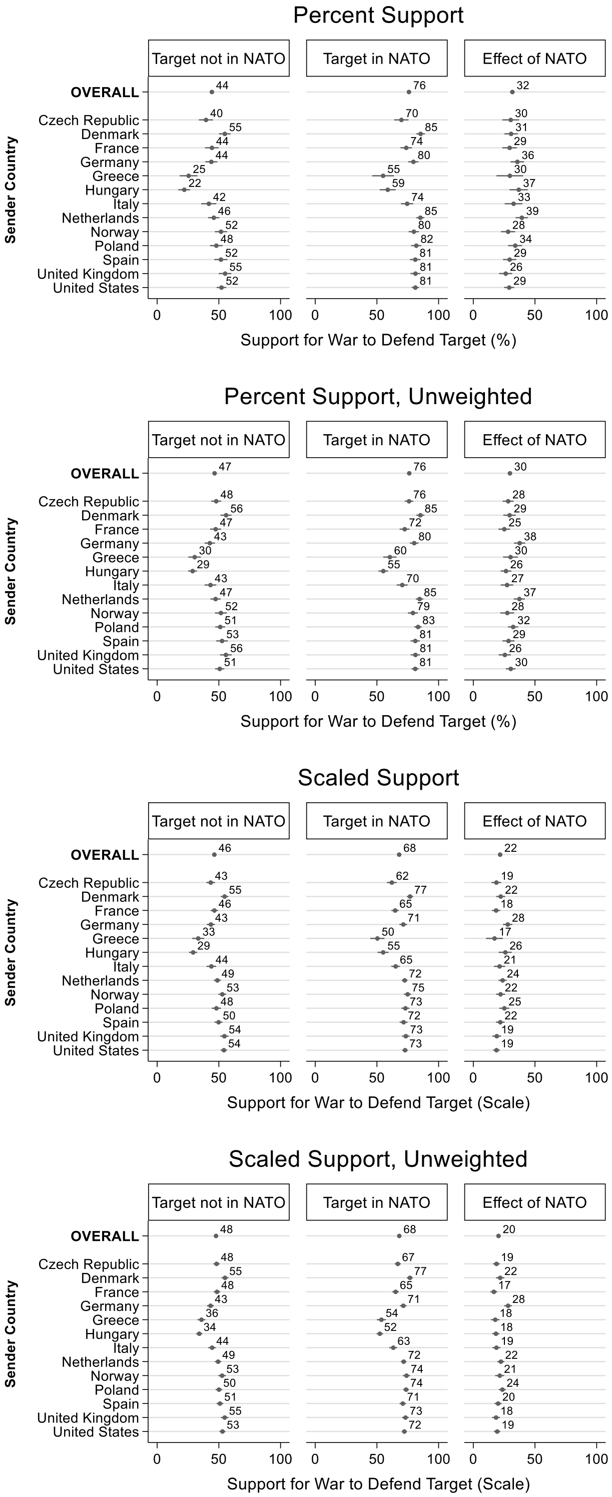

Note: The figure gives equal weight to each target country. Overall was estimated by giving equal weight to each sender country.

Figure S18: Respondents with High Political Interest

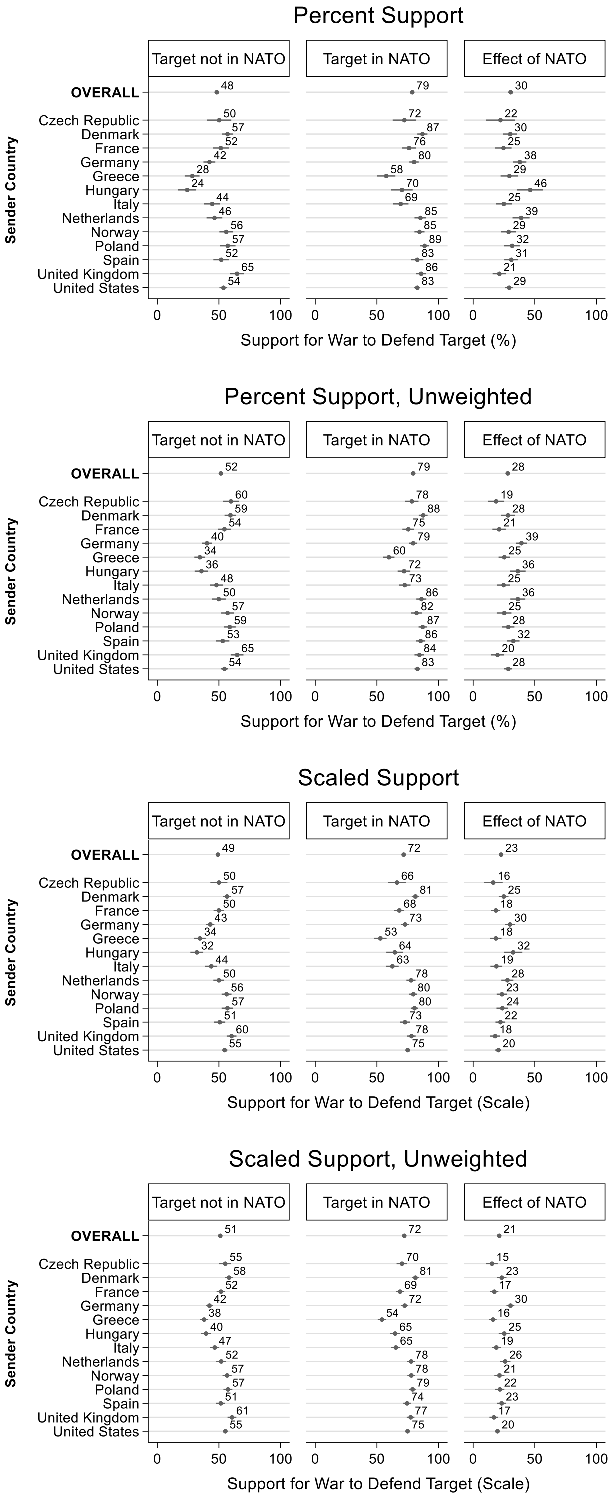

Note: The figure gives equal weight to each target country. Overall was estimated by giving equal weight to each sender country.

Figure S19: Respondents At Least 40 Years Old

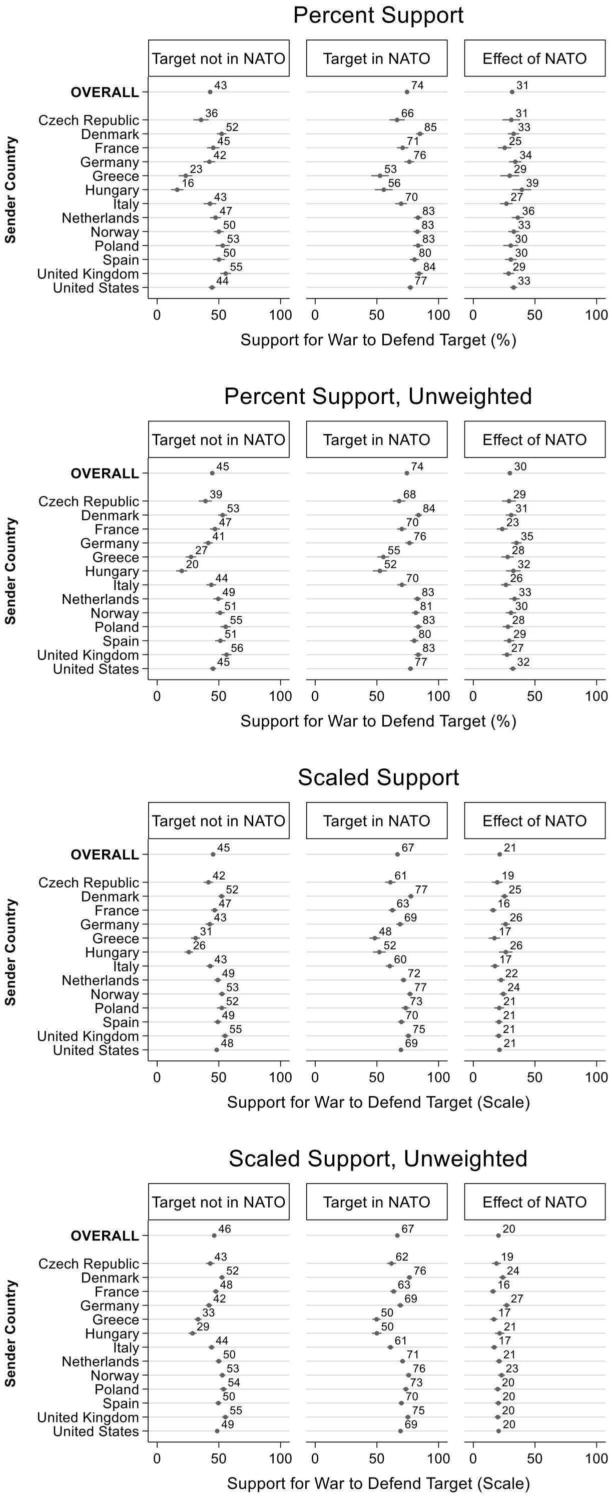

Note: The figure gives equal weight to each target country. Overall was estimated by giving equal weight to each sender country.

Respondents in Non-NATO Sender Countries

Figure S20: Effect of Target Joining NATO

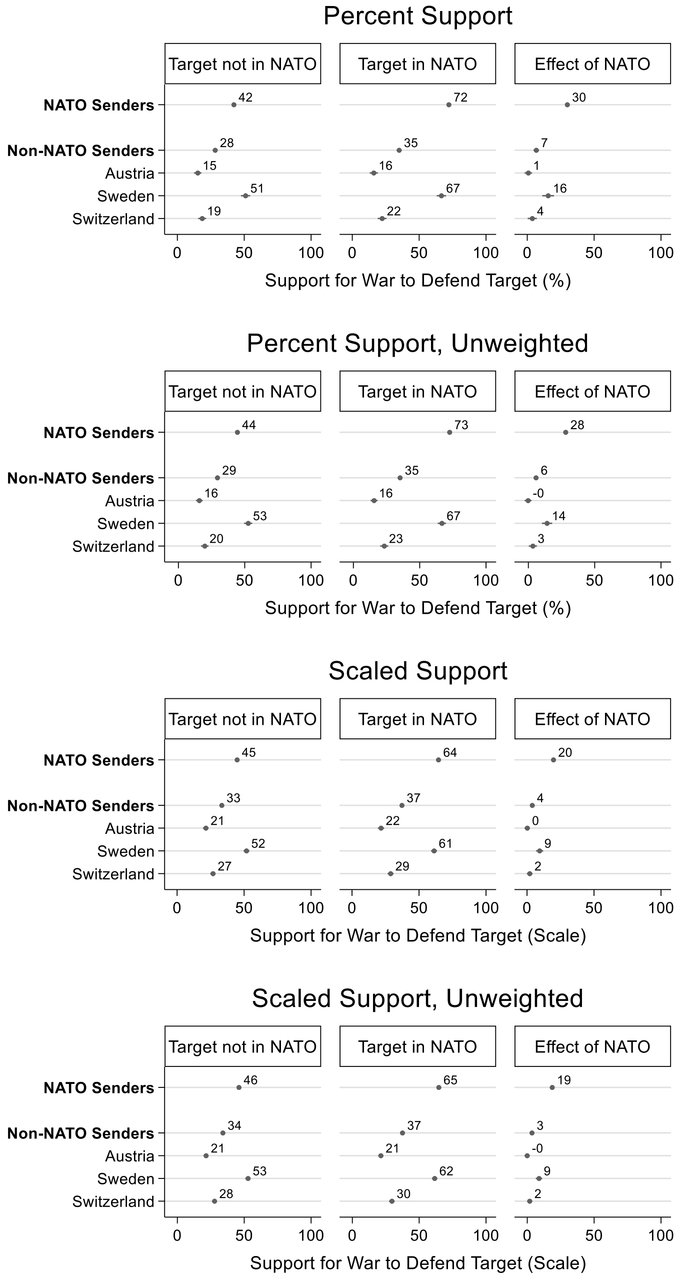

Note: The figure gives equal weight to each target except Sweden, which was excluded from the list of targets. The value for NATO senders gives equal weight to each of the 13 NATO sender countries, and the value for non-NATO senders gives equal weight to each of the 3 non-NATO sender countries.

Figure S21: Effect of Target Joining NATO, By Attitudes about Joining NATO

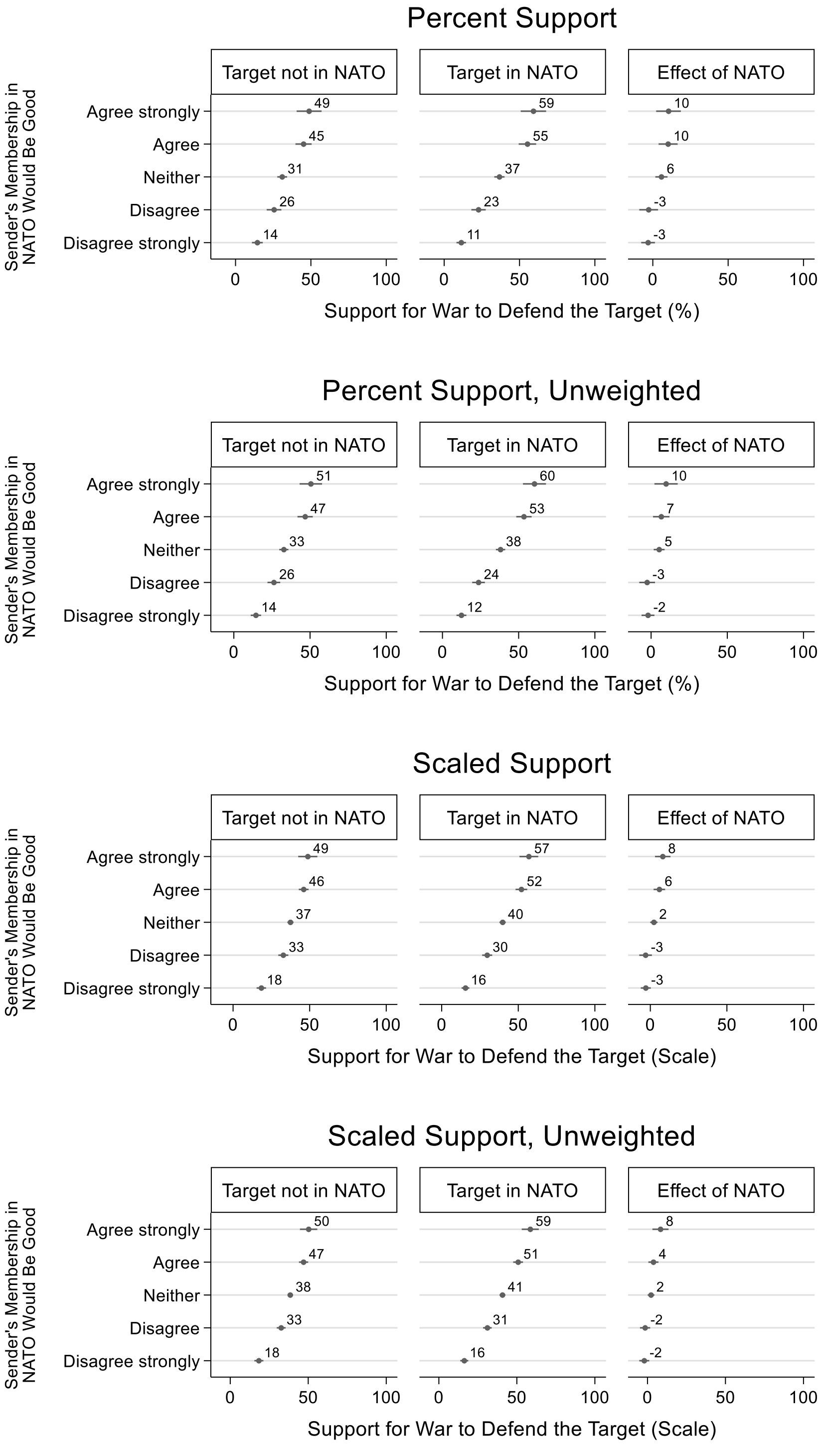

Note: The sample included all targets except Sweden, which was excluded from the list of targets. The figure gives equal weight to each sender country.

III. SUPPORTING TABLES

Summary Statistics for Respondents in NATO Member Countries

Table S1: Number of Respondents in Each of the 13 NATO Member Countries

Country:	Sample Size
Czech Republic	988
Denmark	989
France	996
Germany	957
Greece	996
Hungary	994
Italy	993
Netherlands	989
Norway	987
Poland	995
Spain	997
United Kingdom	999
United States	2,352
Total	14,232

Note: The sample sizes are reported after removing respondents who did not qualify.

Table S2: Gender and Age of Sample in the 13 NATO Member Countries

Country:	Gender (%)		Age (%)		
	Female	Male	18-39	40-59	60+
Czech Republic	50	50	30	38	31
Denmark	51	49	33	34	34
France	52	48	31	34	35
Germany	51	49	30	34	36
Greece	53	47	34	37	28
Hungary	51	49	31	37	31
Italy	52	48	27	37	37
Netherlands	51	49	32	34	34
Norway	50	50	35	34	31
Poland	52	48	33	34	33
Spain	52	48	28	39	33
United Kingdom	51	49	34	34	33
United States	51	49	36	32	32

Note: The statistics are reported after weighting.

Table S3: Education of Sample in the 13 NATO Member Countries

Country:	Education (%)		
	Below upper secondary	Upper secondary or post-secondary non-tertiary	Tertiary
Czech Republic	6	70	24
Denmark	18	41	40
France	18	42	40
Germany	14	55	31
Greece	8	51	41
Hungary	14	57	28
Italy	37	43	20
Netherlands	19	38	42
Norway	17	37	45
Poland	7	60	33
Spain	37	23	40
United Kingdom	18	32	49
United States	8	42	50

Note: The statistics are reported after weighting.

Table S4: Attitudes about NATO Membership in the 13 NATO Member Countries

Country:	Agreement that own country's "membership in NATO is a good thing" (%)				
	Disagree strongly	Disagree	Neither	Agree	Agree strongly
Czech Republic	5	6	20	33	36
Denmark	1	2	13	26	58
France	3	5	27	32	33
Germany	3	4	20	28	46
Greece	9	10	35	29	16
Hungary	3	4	25	28	40
Italy	7	8	28	32	25
Netherlands	2	2	16	40	39
Norway	2	2	13	23	59
Poland	1	1	12	27	59
Spain	4	5	22	36	33
United Kingdom	1	2	17	30	49
United States	3	3	26	33	36

Note: The statistics are reported after weighting.

Summary Statistics for Respondents in Non-NATO Member Countries

Table S5: Number of Respondents in Each of the 3 Non-NATO Member Countries

Country:	Sample Size
Austria	996
Sweden	989
Switzerland	991
Total	2,976

Note: The sample sizes are reported after removing respondents who did not qualify.

Table S6: Gender and Age of Sample in the 3 Non-NATO Member Countries

Country:	Gender (%)		Age (%)		
	Female	Male	18-39	40-59	60+
Austria	51	49	32	35	33
Sweden	50	50	34	33	33
Switzerland	51	49	33	36	32

Note: The statistics are reported after weighting.

Table S7: Education of Sample in the 3 Non-NATO Member Countries

Country:	Education (%)		
	Below upper secondary	Upper secondary or post-secondary non-tertiary	Tertiary
Austria	14	52	34
Sweden	16	39	45
Switzerland	11	44	45

Note: The statistics are reported after weighting.

Table S8: Attitudes about NATO Membership in the 3 Non-NATO Member Countries

**Country:**	**Agreement that own country's "membership in NATO would be a good thing" (%)**				
	Disagree strongly	Disagree	Neither	Agree	Agree strongly
Austria	34	19	32	9	5
Sweden	9	9	27	28	27
Switzerland	20	20	40	15	6

Note: The statistics are reported after weighting.

IV. QUESTIONNAIRE (UNITED STATES)

Note: This questionnaire includes not only questions we preregistered for this article, but also questions that were included for unrelated projects.

Consent

You are invited to participate in a research study of public opinion. You will be asked questions about current economic, social, and political issues. The survey should take about 8 minutes to complete.

There are no risks associated with this study. You will be compensated according to your agreement with the survey company. We cannot and do not guarantee or promise that you will receive any benefits from this study.

If you have read this form and have decided to participate in this project, please understand your participation is voluntary and you have the right to withdraw your consent or discontinue participation at any time without penalty or loss of benefits to which you are otherwise entitled. The alternative is not to participate. Your individual privacy will be maintained in all published and written data resulting from the study.

If you have any questions, concerns or complaints about this research study, its procedures, risks and benefits, you should ask the Protocol Director, Professor Michael Tomz of Stanford University, at (650) 725-4031, email tomz@stanford.edu. If you are not satisfied with how this study is being conducted, or if you have any concerns, complaints, or general questions about the research or your rights as a participant, please contact the Stanford Institutional Review Board (IRB) to speak to someone independent of the research team at (650)-723-2480 or toll free at 1-866-680-2906, or email at irbnonmed@stanford.edu. You can also write to the Stanford IRB, Stanford University, 1705 El Camino Real, Palo Alto, CA 94306. Please save or print a copy of this page for your records.

If you agree to participate in this research, please select "I agree to participate." If you do not agree to participate, the survey will end immediately.

o I agree to participate
o I do not agree to participate

Programming instructions: If respondent selects "I do not agree to participate," end the survey.

—new page—

Screening Questions

Are you a citizen of the United States?
o Yes
o No

Programming instructions: If respondent selects "No," end the survey.

—new page—

Are you eligible to vote in the United States?
o Yes
o No

Programming instructions: If respondent selects "No," end the survey.

—new page—

Below, you will see a series of statements. Please tell us whether you agree or disagree with each statement.

Programming instructions: randomize the order of these items.

2 + 2 = 7
o Agree strongly
o Agree
o Neither agree nor disagree
o Disagree
o Disagree strongly

Please click the "neither agree nor disagree" response
o Agree strongly
o Agree
o Neither agree nor disagree
o Disagree
o Disagree strongly

The year 1910 came before the year 1920
o Agree strongly
o Agree
o Neither agree nor disagree
o Disagree
o Disagree strongly

The use of military force only makes problems worse.
o Agree strongly
o Agree
o Neither agree nor disagree
o Disagree
o Disagree strongly

The United States needs to play an active role in solving conflicts around the world.
o Agree strongly
o Agree
o Neither agree nor disagree
o Disagree
o Disagree strongly

Programming instructions: Confirm that respondent chose "disagree" or "disagree strongly" when prompted that 2+2=7; chose "neither agree nor disagree" when prompted to "click the neither agree nor disagree response"' and choose "agree" or "agree strongly" when prompted that "the year 1910 came before the year 1920." If respondent did not choose these answers, end the survey.

—new page—

Pretreatment Questions

Please specify your gender
o Male
o Female
o Other

In what year were you born?

o 2004
o 2003
…
o 1920

—*new page*—

Were you born in the United States?
o Yes
o No

—*new page*—

Generally speaking, do you think of yourself as a ...
o Republican
o Democrat
o Independent
o Another party, please specify
o No preference

—*new page*—

[If Republican] Would you call yourself a ...
o Strong Republican
o Not very strong Republican

[If Democrat] Would you call yourself a ...
o Strong Democrat
o Not very strong Democrat

[If neither Republican or Democrat] Do you think of yourself as closer to the ...
o Republican Party
o Democratic Party
o Neither party

—*new page*—

In general, do you think of yourself as ...
o Extremely liberal
o Liberal
o Slightly liberal
o Moderate, middle of the road
o Slightly conservative
o Conservative
o Extremely conservative

—new page—

In politics people often talk of "left" and "right". On this scale from 0 (left) to 10 (right), where would you classify your own political views?

o 0 left
o 1
…
o 10 right

—new page—

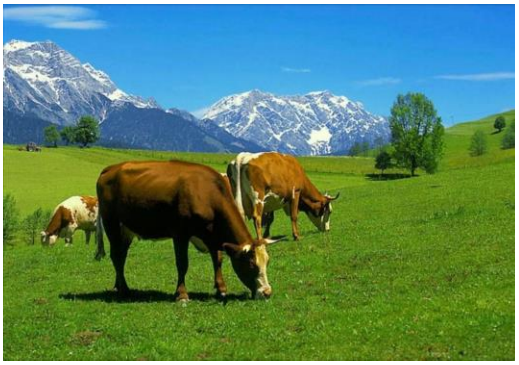

Which one of these is a part of the animal in the picture?
o Fin
o Tail
o Wings
o Beak
o Finger

Programming instructions: If respondent does not select "tail," end the survey.

—new page—

How strongly do you agree or disagree with each of the following statements?

Programming instructions: randomize the order of these items.

Although the media often reports about national and international events and developments, this news is seldom as interesting as the things that happen directly in our own community and neighborhood.
o Agree strongly
o Agree
o Neither agree nor disagree
o Disagree
o Disagree strongly

I feel more like a citizen of the world than of any country.
o Agree strongly
o Agree
o Neither agree nor disagree
o Disagree
o Disagree strongly

I enjoy learning about different cultures.
o Agree strongly
o Agree
o Neither agree nor disagree
o Disagree
o Disagree strongly

The United States has many things to learn from other countries.
o Agree strongly
o Agree
o Neither agree nor disagree
o Disagree
o Disagree strongly

—*new page*—

How strongly do you agree or disagree with each of the following statements?

Programming instructions: randomize the order of these items.

I would rather be a citizen of the United States than of any other country in the world.
o Agree strongly
o Agree
o Neither agree nor disagree
o Disagree
o Disagree strongly

Generally speaking the United States is a better country than most other countries.
o Agree strongly
o Agree
o Neither agree nor disagree
o Disagree
o Disagree strongly

The United States should follow its own interests, even if this leads to conflicts with other nations.
o Agree strongly
o Agree
o Neither agree nor disagree
o Disagree
o Disagree strongly

The U.S. government should just try to take care of the wellbeing of U.S. citizens and not get involved with other nations.
o Agree strongly
o Agree
o Neither agree nor disagree
o Disagree
o Disagree strongly

—new page—

Next, we would like your opinion about some possible international concerns for the United States.

Programming instructions: randomize the order of these items.

Do you think that Russia’s economic and political power is a major threat, a minor threat, or not a threat to the United States?
o Major threat
o Minor threat
o Not a threat

How concerned are you about Russia using military force against the United States?
o Extremely concerned
o Very concerned
o Somewhat concerned
o Not very concerned
o Not at all concerned

—new page—

How strongly do you agree or disagree with each of the following statements regarding the North Atlantic Treaty Organization (NATO) and Russia?

Programming instructions: randomize the order of these items.

The United States’s membership in NATO is a good thing.
o Agree strongly
o Agree
o Neither agree nor disagree
o Disagree
o Disagree strongly

NATO should intervene in Ukraine against the ongoing Russian invasion.
o Agree strongly
o Agree
o Neither agree nor disagree
o Disagree
o Disagree strongly

Russia's invasion of Ukraine represents a threat to all of Europe.
o Agree strongly
o Agree
o Neither agree nor disagree
o Disagree
o Disagree strongly

—new page—

Randomized Treatments

We randomly varied the following features:

The name of target country 1

- *target1* = Finland **–OR–**
- *target1* = Sweden

The name of target country 2

- *target2* = Bosnia **–OR–**
- *target2* = Georgia

Which target country was a member of NATO (*member1* is the membership status of *target1*, and *member2* is the membership status of *target2*)

- *member1* = yes and *member2* = no **–OR–**
- *member1* = no and *member2* = yes

Which of the two targets was presented in the first vignette (if *orderNATO*=t1, present vignette with target1 before vignette with target2. If *orderNATO*=t2, present vignette with target2 before vignette with target1.)

- *orderNATO* = t1 **–OR–**
- *orderNATO* = t2

Text of the Experiment

Please read these facts about NATO:

- The United States is a member of NATO.

- The NATO treaty says that if any member of NATO is attacked, the other members will take all necessary actions, including the use of armed force, to defend their ally.

o Click here after you have read this information carefully.

—*new page*—

Programming instructions: if orderNATO=t1, present vignette with target1 before vignette with target2. If orderNATO=t2, present vignette with target2 before vignette with target1.)

Vignette with target1

If target1 = Finland, display: The map below shows **the country of Finland**, which is located in Europe.

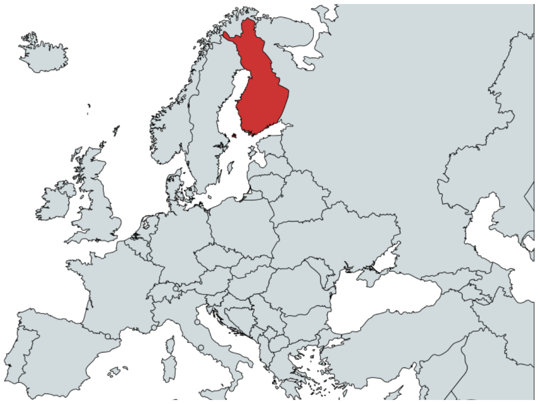

If target1 = Sweden, display: The map below shows **the country of Sweden**, which is located in Europe.

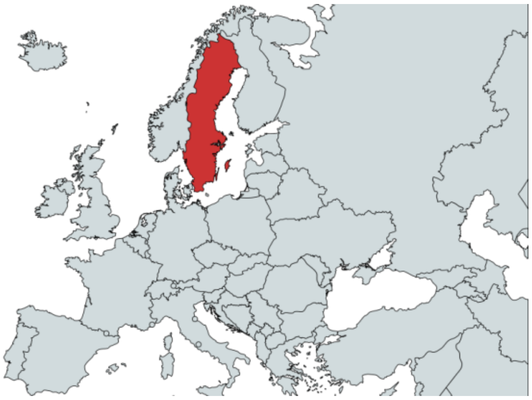

There is much discussion about whether **the country of ${e://Field/target1}** will become a member of NATO.

If member1 = yes, display: Suppose that ${e://Field/target1} **becomes a member** of NATO, and then Russia attacks ${e://Field/target1}. In that situation, do you think the United States should or should not use military force to defend ${e://Field/target1}?

o Definitely should
o Probably should
o Probably should not
o Definitely should not

If member1 = no, display: Suppose that ${e://Field/target1} **does not become a member** of NATO, and then Russia attacks ${e://Field/target1}. In that situation, do you think the United States should or should not use military force to defend ${e://Field/target1}?

o Definitely should
o Probably should
o Probably should not
o Definitely should not

—*new page*—

Vignette with target2

If target2 = Finland, display: The map below shows **the country of Bosnia**, which is located in Europe.

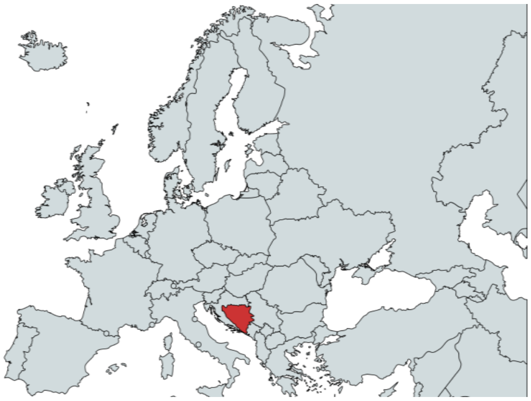

If target2 = Georgia, display: The map below shows **the country of Georgia**, which is located in Europe.

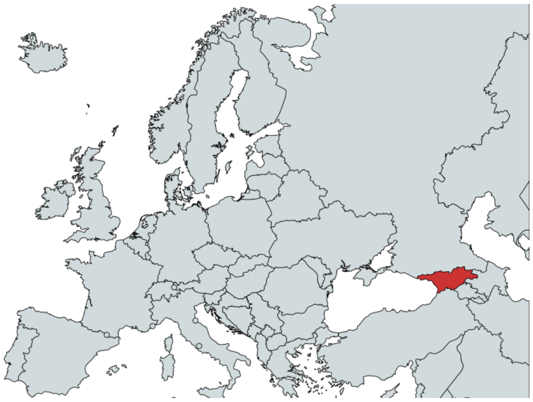

There is much discussion about whether **the country of ${e://Field/target2}** will become a member of NATO.

If member2 = yes, display: Suppose that ${e://Field/target2} **becomes a member** of NATO, and then Russia attacks ${e://Field/target2}. In that situation, do you think the United States should or should not use military force to defend ${e://Field/target2}?

- o Definitely should
- o Probably should
- o Probably should not
- o Definitely should not

If member2 = no, display: Suppose that ${e://Field/target2} **does not become a member** of NATO, and then Russia attacks ${e://Field/target2}. In that situation, do you think the United States should or should not use military force to defend ${e://Field/target2}?

- o Definitely should
- o Probably should
- o Probably should not
- o Definitely should not

—*new page*—

Additional Background Questions

How interested would you say you are in politics? Are you:
o Extremely interested
o Very interested
o Moderately interested
o Slightly interested
o Not at all interested

—*new page*—

Are you of Hispanic or Latino origin?
o No
o Yes, Mexican, Mexican American, Chicano
o Yes, Puerto Rican
o Yes, Cuban
o Yes, another Hispanic, Latino, or Spanish origin (please specify)

Which of the following best describes your race (mark all that apply)?
▢ White
▢ Black or African American
▢ American Indian or Alaska Native
▢ Asian
▢ Native Hawaiian or Other Pacific Islander
▢ Some other race (please specify)

—*new page*—

Now we would like to ask you some final questions about your living conditions. Which of these options best describes your situation (in the last seven days)?
o Paid employee (including temporary leave of absence due to maternity/paternity, accident, illness or vacation)
o Self-employed (e.g. freelancer, independent contractor, or family-owned business)
o Student (excluding employer-sponsored education)
o Unemployed, actively searching for a job
o Unemployed, not actively searching
o Chronic illness or permanent disability
o Retired
o Working at home, caring for children or others

—*new page*—

What is the highest level of education you have completed?
o Did not graduate from high school
o High school graduate
o Some college, but no degree (yet)
o 2-year college degree
o 4-year college degree
o Postgraduate degree (MA, MBA, MD, JD, PhD, etc.)

—*new page*—

Thinking back over the last year, what was your family's annual income?

This information is very important for the validity of the study. Your information will be kept strictly confidential and anonymous.

o Less than $10,000
o $10,000 - $19,999
o $20,000 - $29,000
o $30,000 - $39,999
o $40,000 - $49,999
o $50,000 - $59,999
o $60,000 - $69,999
o $70,000 - $79,999
o $80,000 - $99,999
o $100,000 - $119,999
o $120,000 - $149,999
o $150,000 or more
o Prefer not to say

—*new page*—

Which of the following statements comes closest to how you feel about your household’s income nowadays?
o With our current income, we live comfortably
o With our current income, we make ends meet
o With our current income, we have difficulties
o With our current income, we have major difficulties

—new page—

In what state do you currently reside?

o Alabama
o Alaska
o Arizona
o Arkansas
o California
o Colorado
o Connecticut
o Delaware
o District of Columbia
o Florida
o Georgia
o Hawaii
o Idaho
o Illinois
o Indiana
o Iowa
o Kansas
o Kentucky
o Louisiana
o Maine
o Maryland
o Massachusetts
o Michigan
o Minnesota
o Mississippi
o Missouri
o Montana
o Nebraska
o Nevada
o New Hampshire
o New Jersey
o New Mexico
o New York
o North Carolina
o North Dakota
o Ohio
o Oklahoma
o Oregon
o Pennsylvania
o Puerto Rico
o Rhode Island
o South Carolina
o South Dakota
o Tennessee
o Texas
o Utah
o Vermont
o Virginia

- o Washington
- o West Virginia
- o Wisconsin
- o Wyoming
- o I do not reside in the United States

What is the city/town in which you reside? _______
